# Estimation of pulmonary function from time‐resolved dynamic chest radiography using machine learning in patients with respiratory disease

**DOI:** 10.1002/acm2.70717

**Published:** 2026-07-27

**Authors:** Takehiro Shiinoki, Yuki Yuasa, Tsunahiko Hirano, Maki Asami‐Noyama, Kazuto Matsunaga, Hidekazu Tanaka

**Affiliations:** ^1^ Department of Radiation Oncology Graduate School of Medicine Yamaguchi University Ube Yamaguchi Japan; ^2^ Department of Radiological Technology Yamaguchi University Hospital Ube Yamaguchi Japan; ^3^ Department of Respiratory Medicine and Infectious Disease Graduate School of Medicine Yamaguchi University Ube Yamaguchi Japan

**Keywords:** dynamic chest radiography, machine learning, pulmonary function, radiomics, respiratory motion map

## Abstract

**Background:**

Pulmonary function tests (PFTs), particularly spirometry, are the reference standard for assessing airflow limitation in respiratory diseases such as chronic obstructive pulmonary disease (COPD) and interstitial pulmonary disease. However, spirometry requires substantial patient cooperation and may be unreliable in children, the elderly, and patients with cognitive impairment, and its use was further limited during the COVID‐19 pandemic. Dynamic chest radiography (DCR), which captures sequential thoracic images during respiration at low radiation dose, has emerged as a promising modality for evaluating respiratory dynamics, but its potential to quantitatively estimate pulmonary function through radiomic analysis remains insufficiently explored.

**Purpose:**

This study aimed to determine the potential of radiomic features of the lung on DCR to predict pulmonary function (FEV_1_, forced expiratory volume in the first second; FVC, forced vital capacity) and to classify patients at high risk (FEV_1_/FVC).

**Methods:**

We retrospectively analysed data from 151 patients. The DCRs at end‐inspiration (EI), end‐expiration (EE), and the respiratory phase of maximum variation in the lung area from EI to EE (insp2exp_vmax_) or from EE to EI (exp2insp_vmax_) were defined based on the lung area. A respiratory motion map was also calculated. To combine the defined DCR and respiratory motion map, feature extraction was performed, followed by the least absolute shrinkage and selection operator (LASSO). Predictive regression and classification models with various radiomic feature combinations (nos. 1–6) were constructed for pulmonary function. Pearson's correlation coefficients (R) were calculated for FEV_1_ and FVC, and the area under the curve (AUC) was calculated for FEV_1_/FVC. Our predictive models were compared using the conventional formula.

**Results:**

We constructed a predictive regression and classification model for FEV_1_, FVC, and FEV_1_/FVC ratio using DCR images and a respiratory motion map. The model accuracy with DCR at each respiratory phase and the respiratory motion map‐based radiomic features was better than that of the conventional method.

**Conclusions:**

In this single‐center retrospective study, radiomic features extracted from DCR at multiple respiratory phases combined with respiratory motion maps showed promise for estimating pulmonary function, outperforming conventional demographic‐based prediction. External validation in multi‐center cohorts is warranted before clinical translation.

## 1 INTRODUCTION

Assessment of lung function is critical for diagnosing lung diseases such as asthma, chronic obstructive pulmonary disease (COPD), and interstitial pulmonary disease (IP). Pulmonary function tests (PFTs), including spirometry, diffusing capacity measurement, and lung volume assessment, are widely used to evaluate lung function. Among these, spirometry is the most commonly performed test, quantifying airflow by measuring how effectively a person inhales or exhales a specific volume of air over a given period. Its key indices — forced expiratory volume in the first second (FEV_1_) and forced vital capacity (FVC)—serve as the reference standard for detecting airflow limitation, although spirometry alone does not comprehensively assess all aspects of pulmonary function. These indices are essential for diagnosing and managing patients with respiratory disease. In general, a threshold of 70% for the FEV_1_/FVC ratio is used to differentiate between normal and obstructive ventilatory patterns.^1^ However, patient cooperation is essential, and obtaining reliable data may be challenging, especially in children, elderly patients, and those with cognitive impairment. Testing was also limited during the COVID‐19 pandemic due to concerns about the potential for PFTs to spread infections among healthcare workers and patients. Furthermore, spirometry is associated with diagnostic difficulties. Moreover, a challenge arises from the potential misinterpretation of spirometry measurements by primary care providers, which can lead to misdiagnoses.^2^ In such instances, there is a tendency for primary care providers to overdiagnose COPD, with general practitioners suggesting an inaccurate COPD diagnosis in approximately one‐third of cases.^2^, ^3^

Kristensen et al.^4^ developed a prediction model for pulmonary function using machine learning and demography data. Miyoshi et al.^5^ also developed a prediction model for pulmonary function using forced oscillometry in patients with asthma, COPD, and IP. They developed a predictive model by applying the data acquired from conventional PFTs.

Radiomics is an emerging research area that involves extracting complex features from medical imaging data, such as computed tomography (CT) and magnetic resonance imaging scans. It is gaining increasing interest because of its potential to predict tumour prognosis, assist in pathological analysis, and forecast adverse effects using advanced features and artificial intelligence technologies.^6^, ^7^ Several researchers have developed prediction models for pulmonary function with CT images using deep learning and radiomics analysis.^8^, ^9^

Imaging techniques such as chest radiography and CT continue to be essential for characterising lung diseases. Dynamic chest radiography (DCR) is an innovative time‐sequential radiographic imaging method capable of visualising lung and diaphragm motion during tidal or guided deep breathing.^10^ This technique quantifies diaphragm motion as a marker of lung function. A previous study reported a difference in diaphragm motion between patients with COPD and healthy controls.^11^ Nonetheless, the full potential of DCR remains untapped because changes in lung area and pixel values have not been thoroughly evaluated. Therefore, the application of radiomic analysis to DCR has the potential to enhance the diagnostic value of DCR examination of the lungs.

Although spirometry remains the reference standard for assessing airflow limitation, it requires substantial patient cooperation, trained operators, and specialised equipment. Reliable measurements are particularly challenging to obtain in children, elderly patients, and those with cognitive impairment, and testing may be restricted in contexts such as infection control during the COVID‐19 pandemic. In contrast, DCR can be performed during tidal or guided deep breathing without requiring active patient cooperation and is increasingly integrated into routine clinical chest imaging. Furthermore, unlike spirometry, which provides only global lung function measurements, DCR has the potential to provide spatially resolved information about regional lung mechanics, reflecting the heterogeneous nature of obstructive and restrictive lung diseases. DCR thus represents a promising complementary tool for lung function assessment in clinical populations where spirometry is unreliable, difficult to perform, or difficult to interpret. Various other imaging modalities have been investigated for regional lung function assessment, including hyperpolarised gas MRI, Fourier decomposition MRI, and dynamic CT, which provide detailed physiological information but are limited by high cost, specialised equipment requirements, or substantial radiation exposure. DCR utilises widely available digital radiography equipment with comparatively low radiation exposure, enabling real‐time visualisation of lung dynamics during tidal or guided breathing, which makes it an attractive option for widespread clinical application.

This study aimed to determine the potential of radiomic features of the lung on DCR to predict pulmonary function (FEV_1_ and FVC) and classify patients at high risk (FEV_1_/FVC).

## 2 MATERIALS AND METHODS

### 2.1 Patients’ data and data acquisition

In total, 151 patients who underwent DCR between January 2023 and April 2024 were retrospectively included in this study. The exclusion criteria were as follows: (1) patients other than those with asthma, COPD, IP, and ACOS (asthma‐COPD overlap syndrome); (2) patients with a pacemaker; and (3) a discrepancy of more than one month between the dates of DCR and spirometer inspections. After applying these criteria, 124 patients were evaluated retrospectively.

Table 1 summarizes patient characteristics. This single‐centre retrospective study was approved by the Institutional Review Board of Yamaguchi University Hospital (approval no. 2024–081). Written informed consent was waived because of the retrospective design. This retrospective study was performed according to the relevant institutional guidelines and regulations.

**TABLE 1:** Patients and clinical characteristics.

	Training cohort	Test cohort
Number of patients	86	38
	Males (*n* = 50)	Males (*n* = 18)
	Females (*n* = 36)	Females (*n* = 20)
Age	66.4 ± 12.6	64.5 ± 16.2
Smoking (%Yes)	64.0	60.5
Asthma	43	16
COPD	16	11
IP	25	11
ACOS	2	0
PFT		
FVC (L: mean ± S.D.)	3.0 ± 0.9	2.8 ± 0.8
FEV_1_ (L: mean ± S.D.)	2.2 ± 0.6	1.9 ± 0.6
FEV_1_/FVC (%: mean ± S.D.)	71.6 ± 15.7	68.0 ± 16.6

Abbreviations: ACOS, asthma‐COPD Overlap syndrome; COPD, chronic obstructive pulmonary disease; FEV_1_, forced expiratory volume in 1 s; FVC, forced vital capacity; IP, interstitial pneumonia; PFT, pulmonary function test.

For all patients, anteroposterior DCR images were acquired in the standing or sitting position using a dynamic flat‐panel detector imaging system (Konica Minolta, Inc., Tokyo, Japan). The acquisition parameters are summarised in Table 2. The dynamic image data, captured at 15 frames per second, were synchronised with the pulsed x‐ray. Each examination lasted 20 s, yielding 300 frames per acquisition at this 15 frames‐per‐second rate, and all 300 frames were analysed to derive the lung‐area time curve and the respiratory motion map (Figure S1). As in modern fluoroscopy, the chest DCR in this study utilised pulsed x‐rays to prevent excessive exposure of the participants to radiation. During DCR acquisition, patients were instructed to perform deep breathing maneuvers (maximal inspiration to maximal expiration) under automated voice guidance (Figure S1), approximating the respiratory excursions used in spirometry, although without the forced expiratory effort required for FEV_1_ measurement. The estimated effective dose per DCR examination was approximately 0.2 mSv,^10^ which is substantially lower than that of CT pulmonary angiography and approximately one‐tenth of that of lung ventilation‐perfusion scintigraphy.

**TABLE 2:** Acquisition parameters for DCR.

Parameter	Value
Tube voltage (kV)	85–110
Tube current (mA)	80–160
Pulse duration (ms)	4–8
mAs per pulse	0.32–1.28
Frame rate (fps)	15
Acquisition time (s)	20
Total frames per acquisition	300
Source‐to‐image distance (cm)	200
Additional filtration	0.2 mm Cu
Pixel size (µm)	400 × 400
Image area (mm)	424.8 × 424.8
Gray‐level depth (bits)	16
Estimated effective dose (mSv)	≈ 0.2

Additionally, PFTs were performed using spirometry (CHESTAC‐8800 DN type, Chest Ltd., Tokyo, Japan), following the recommendations of the American Thoracic Society/European Respiratory Society.^12^ FEV_1_, FVC, and FEV_1_/FVC were recorded.

### 2.2 Segmentation and definition of the respiratory phase for DCR

**FIGURE 1:**
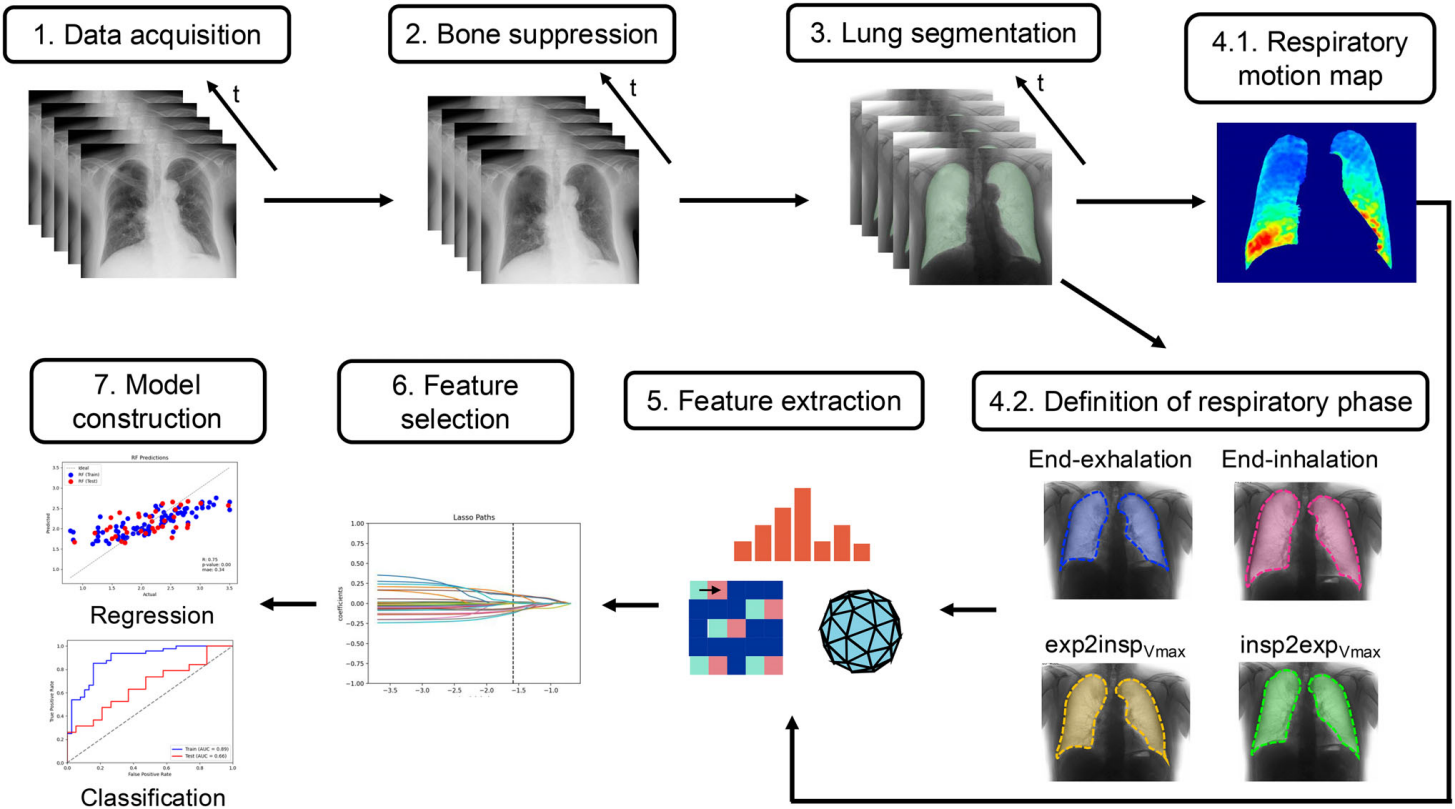
The schematic of this study design. DCR was acquired. The DCR image was processed with bone suppression. For DCR, lung segmentation was performed using deep learning. The respiratory motion map was calculated using optical flow. Additionally, each respiratory phase of DCR (EE, EI, exp2insp_vmax_, and insp2exp_vmax_) was defined based on the calculated lung area. Feature extraction was performed for DCR at each respiratory phase and the respiratory motion map. The least absolute shrinkage and selection operator (LASSO) was used to select the optimised feature, and the prediction models were constructed for PFT.

Figure 1 illustrates the strategies used in this study. The DCR images were processed with bone suppression using the manufacturer's proprietary software (KINOSIS, Konica Minolta, Inc., Tokyo, Japan), which applies a deep learning‐based algorithm to suppress thoracic skeletal structures while preserving lung parenchymal information. Radiomic features were subsequently extracted from the bone‐suppressed for‐presentation images. Although for‐processing (raw) images preserves quantitative pixel‐value information, the for‐presentation images used in this study undergo additional non‐linear processing (e.g., gradation curve adjustment, edge enhancement) applied by the manufacturer's image processing pipeline. The processed images were then converted into DICOM multi‐frame files containing 15 frames each. For the DCR, lung segmentation was performed using deep learning.^13^ All segmentation results were manually reviewed by a respiratory physician, a radiation technologist, and a medical physicist. All necessary modifications were made. Lung areas were calculated for all DCRs. The DCRs at end‐inspiration (EI) and end‐expiration (EE) were defined based on the calculated lung area. Furthermore, the variation in lung area over time was calculated, allowing the respiratory phase of the maximum variation in lung area from EI to EE (insp2expvmax; where “2” denotes “to,” that is, the respiratory phase from inspiration to expiration at maximum lung area variation rate) or from EE to EI (exp2inspvmax) to be defined. A detailed definition of the respiratory phase is provided in Figure S2.

### 2.3 Eulerian respiratory motion map derived from dense optical flow

The respiratory motion map was computed in an Eulerian (image‐space) framework as follows. For each pair of consecutive DCR frames, *I_t_* and *I*_t+1_, dense optical flow vectors V t(p) = (u t(p), w t(p) were estimated at every pixel location p using the Farnebäck algorithm^14^ (OpenCV implementation; pyramid scale = 0.5, pyramid levels = 3, window size = 15, iterations = 3, polynomial neighbourhood = 5, polynomial sigma = 1.2). The magnitude of each flow vector, ||*v* *t*(*p*)|| = √(*u* *t*(*p*)^2^ + *w* *t*(*p*)^2^), was then accumulated across all consecutive frame pairs at each fixed pixel location: *M*(*p*) = Σ ||*v* *t*(*p*)|| (*t* = 1 to *T*−1), where T is the total number of frames in the sequence. The resulting scalar field *M*(*p*) was masked using the lung region defined at EI (as defined in the Segmentation subsection above) to yield the final respiratory motion map used for radiomic feature extraction. This Eulerian formulation characterises the spatial distribution of cumulative motion magnitude observed at each anatomical location during the respiratory cycle, analogous to regional ventilation maps reported in prior DCR studies.^15^

The underlying rationale for using the respiratory motion map to predict pulmonary function is that regional lung parenchymal motion reflects regional ventilation. In regions affected by airway obstruction, air trapping, or fibrosis, local parenchymal displacement during breathing is expected to be reduced or spatially heterogeneous. By extracting radiomic features from the respiratory motion map, we aimed to characterise the texture and distributional patterns of regional lung dynamics, hypothesising that these features correlate with global PFT indices such as FEV_1_ and FVC.

### 2.4 Radiomic feature extraction for DCRs, the respiratory motion map, and reduction

Lung segmented regions at EI, EE, insp2expvmax, and exp2inspvmax were used to extract the radiomic features using the open‐source software Pyradiomics Version 3.7.1 in Python.^16^ In total, 851 radiomic features were extracted for DCR at each respiratory phase, including shape, first‐order, feature shape, Gray Level Co‐occurrence Matrix, Gray Level Run Length Matrix, Gray Level Size Zone Matrix, Neighbouring Gray Tone Difference Matrix, Gray Level Difference Matrix, and wavelet filter. The Image Biomarker Standardisation Initiative defined the feature extraction matrices and formulas.^17^ The primary settings for radiomic feature extraction were as follows: the bin width of the feature extraction parameters was set to 30, as this resulted in high reproducibility of radiomic features and model performance.^18^ The Resample Pixel Spacing was set to 1 × 1 × 1 mm^3^.^19^ Similarly, the respiratory motion features, excluding shape features, were extracted from the respiratory motion map. To prevent multicollinearity, feature selection was performed using a Spearman redundancy filter (|*ρ*| > 0.80; that is, features with pairwise absolute correlation exceeding 0.80 were removed), as reported in previous studies.^20^, ^21^ Regression was performed using the least absolute shrinkage and selection operator (LASSO) to select the optimised feature subset to construct the models.

### 2.5 Bootstrap stability selection

To assess the robustness of the selected radiomic features to perturbations of the training sample, we performed a post‐hoc bootstrap stability‐selection analysis.^22^ The complete three‐stage feature‐selection pipeline (Spearman redundancy filter at |*ρ*| > 0.80 → Pearson correlation–with‐target prefilter at |*r*| > 0.20 for regression targets (FEV_1_ and FVC), or ANOVA F‐test prefilter at *p* < 0.05 for the disease‐classification target → LASSO regression or L1‐penalised multinomial logistic regression with internal 10‐fold cross‐validation) was applied to 200 bootstrap resamples for each radiomic‐feature combination and each clinical target. Features selected in ≥ 80 % of bootstrap iterations were defined as robust. The complete list of robust features is provided in Tables S1, S2, and S3, Figures 4, and S3.

### 2.6 Construction of a predictive model for PFT and performance evaluation

As DCR yields sequential data, we developed several predictive models by combining various DCR phases with a respiratory motion map. Table 3 shows the combination of the radiomic features of DCR at each respiratory phase and the respiratory motion map. The patient data were split 70:30 for model training and testing, respectively. The data were randomly divided at each feature selection step, and the model was constructed. The number of initial radiomic features prior to correlation filtering and LASSO selection varied by combination: single‐phase models (nos. 1 and 2) used 851 features, two‐phase models (no. 3) used 1702 features, four‐phase models (no. 4) used 3404 features, and models incorporating the respiratory motion map (nos. 5 and 6) included an additional 837 features (851 features excluding shape features). Computational time for feature extraction and model training scaled approximately linearly with the number of input features; however, LASSO regularisation substantially reduced the final model dimensionality, and total processing time per patient remained under five minutes for all combinations on a standard workstation.

**TABLE 3:** Radiomic feature combinations of DCR at each phase and a respiratory motion map for prediction model construction.

No.	Radiomic feature combinations
1	EI
2	EE
3	EI + EE
4	EI + EE + insp2exp_vmax_ + exp2insp_vmax_
5	EI + EE + RM
6	EI + EE + insp2exp_vmax_ + exp2insp_vmax_ + RM

Abbreviations: EE, end expiration; EI, end inspiration; RM, respiratory motion map.

### 2.7 Predictive regression model of FEV_1_ and FVC

Predictive regression models were constructed using the random forest algorithm to predict FEV_1_ and FVC in the training cohort.^4^ Across all analyses, LASSO was used as the feature‐selection mechanism (retaining features with non‐zero coefficients), and the downstream regressor was chosen to suit the resulting feature space: random forest was used for the primary single‐split analysis, four algorithms (linear, ridge, LASSO, random forest) were compared in the multi‐seed sensitivity analysis described below, and ordinary linear regression was used for the parsimonious Universal Minimal model on the basis that linear models match or outperform more complex learners when the feature space is small. To exclude outliers (mismeasured values) in spirometry data, a *Z*‐score for FEV_1_/FVC was calculated, and values ≥ 2 were removed (*n* = 4). The hyperparameters were optimised for the training cohort using a 10‐fold cross‐validation and the Optuna algorithm to prevent overfitting.^23^ The performance of the predictive regression models was evaluated using the mean absolute error (MAE). The Pearson correlation coefficients (R) were calculated to evaluate the relationship between measured and predicted FEV_1_ and FVC. The trained models were then assessed using an independent testing cohort. Furthermore, predictive regression models constructed using DCR and respiratory motion map‐based radiomic features were compared with conventional predictive models derived from published reference equations that estimate FEV_1_ and FVC using linear regression based on height, age, and sex.^24^, ^25^

### 2.8 Multi‐seed multi‐algorithm sensitivity analysis

To complement the primary 70: 30 train–test evaluation, the predictive performance of each of the six published feature combinations was re‐evaluated over 50 independent random 70: 30 train–test splits and four regression algorithms—ordinary linear regression, ridge regression, LASSO, and random forest—to characterise the variability of test‐cohort Pearson R and MAE with respect to both random sampling and learning algorithm. We additionally constructed a parsimonious “Universal Minimal” model using only the two radiomic features that emerged as universally robust across both FEV_1_ and FVC in the bootstrap stability analysis (original_shape_MajorAxisLength_EI and original_shape_Elongation_EI). Performance is reported as the mean test Pearson R with 95% confidence interval over the 50 splits.

### 2.9 Classification model of FEV_1_/FVC over 70%

Classification models for FEV_1_/FVC > 70% were constructed using logistic regression for the training cohort. The synthetic minority oversampling technique (SMOTE) was used to improve the data imbalance in the training cohort.^26^ Similar to the predictive regression model described above, the hyperparameters were optimised by a 10‐fold cross‐validation using the Optuna algorithm. The performance of the classification model was evaluated using the area under the curve (AUC) of the receiver operating characteristic curve in a 10‐fold cross‐validation. Subsequently, the trained models were assessed using an independent testing cohort. The accuracy, sensitivity, specificity, positive predictive value (PPV), and negative predictive value (NPV) were calculated to evaluate the classification model comprehensively. Furthermore, predictive classification models using DCR and respiratory motion map‐based radiomic features were compared with the conventional model.

### 2.10 Multi‐class classification of underlying respiratory disease

Beyond the binary FEV_1_/FVC > 70 % classifier, we evaluated whether DCR radiomic features can directly classify the underlying respiratory disease into three diagnostic categories—asthma, chronic obstructive pulmonary disease (COPD), and interstitial pneumonia (IP). Of the 124 patients, 122 had a definitive single‐disease diagnosis recorded (asthma, *n* = 59; COPD, *n* = 27; IP, *n* = 36); two patients with asthma–COPD overlap syndrome were excluded from this analysis. The radiomic feature‐selection pipeline (Spearman redundancy filter → ANOVA F‐test prefilter at *p* < 0.05 → L1‐penalised multinomial logistic regression with internal 10‐fold cross‐validation) was applied as described in the bootstrap stability section. For prediction, four classification algorithms — multinomial logistic regression (unpenalised), L2‐penalised (ridge) and L1‐penalised (LASSO) logistic regression with cross‐validated regularisation strength, and random forest — were compared over 50 stratified random 70: 30 train–test splits, with class_weight = “balanced” to mitigate class imbalance. Predictive performance was reported as macro‐averaged one‐vs‐rest ROC AUC, accuracy, and macro F1.

## 3 RESULTS

### 3.1 Predictive regression model of FEV_1_ and FVC

Table 4 presents the Pearson correlation coefficients and MAE for each prediction model in relation to FEV_1_. For the training cohort, the Pearson correlation coefficient was 0.67 for the conventional method, with coefficients ranging from 0.79 to 0.97 for each radiomic feature combination. The MAE was 0.48 L for the conventional method, with MAEs ranging from 0.19 to 0.35 L for each radiomic feature combination.

**TABLE 4:** The Pearson correlation coefficient and MAE for each FEV_1_ predictive model.

	Train cohort		Test cohort	
No.	R	MAE	R	MAE
conventional	0.67	0.48	0.57	0.44
1	0.79	0.35	0.68	0.33
2	0.86	0.31	0.47	0.38
3	0.84	0.28	0.62	0.32
4	0.97	0.19	0.81	0.29
5	0.93	0.23	0.70	0.31
6	0.93	0.24	0.65	0.33

Abbreviations: MAE, mean absolute error; R, Pearson correlation coefficient.

**FIGURE 2:**
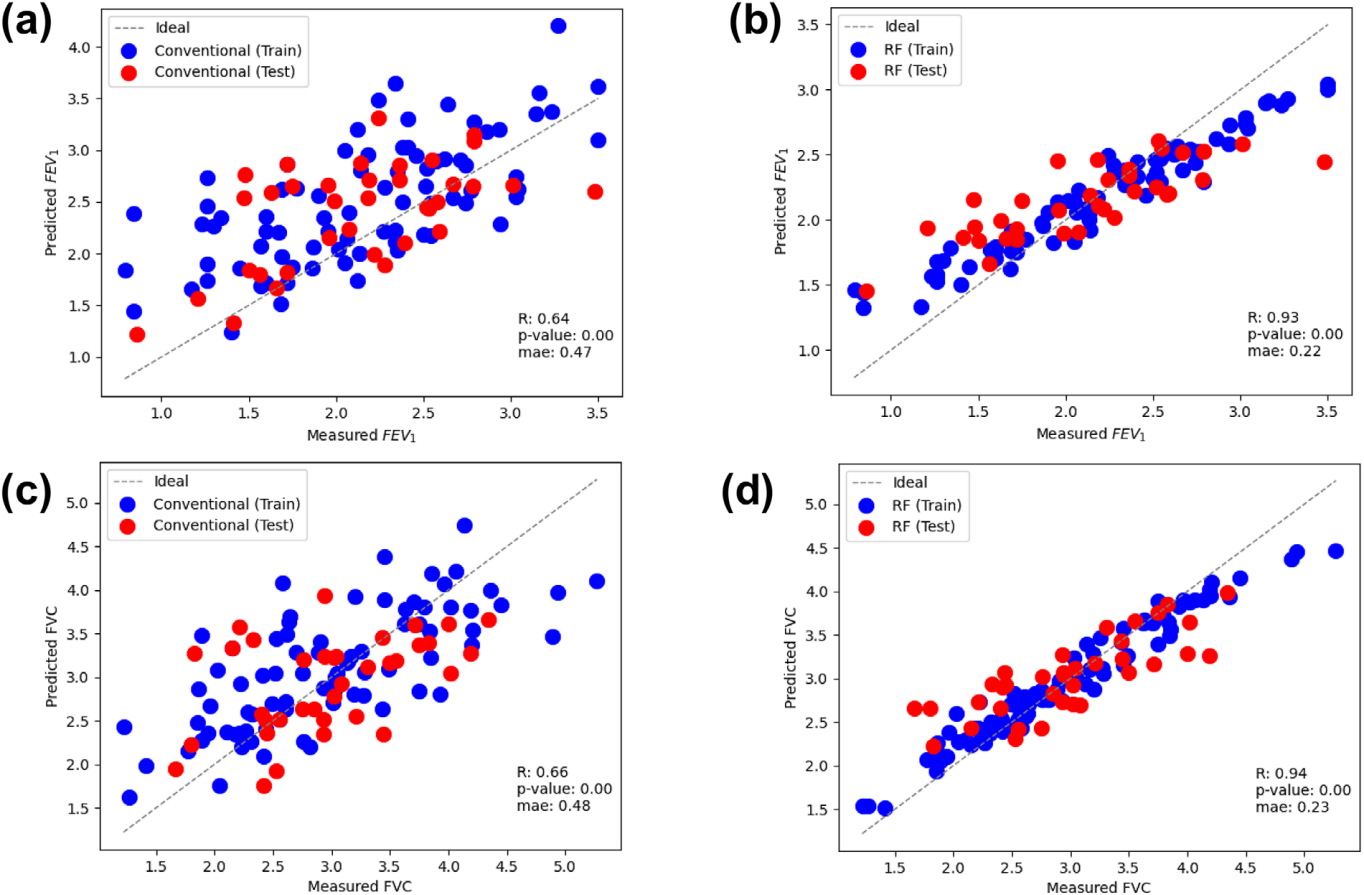
Relationship between predicted and actual FEV_1_ in radiomic feature combination no. 4. (a) For the conventional prediction method, the predicted and actual FEV_1_ demonstrated a moderate correlation for all data [*R* = 0.64, (*p* < 0.05)]. (b) For the random forest (RF) prediction model, the predicted and actual FEV_1_ demonstrated a strong correlation for all data [*R* = 0.93, (*p* < 0.05)]. The MAEs were 0.47 and 0.22 for the conventional and RF prediction models for FEV_1_, respectively. In radiomic feature combination no. 5, the relationship between predicted and measured FVC was evaluated. (c) For the conventional prediction method, the predicted and measured FVC demonstrated a moderate correlation for all data [*R* = 0.66, (*p* < 0.05)]. (d) For the random forest (RF) prediction model, the predicted and measured FVC demonstrated a strong correlation for all data [*R* = 0.94, (*p* < 0.05)]. The MAEs were 0.48 and 0.23 for the conventional and RF prediction models for FVC, respectively.

For the test cohort, the Pearson correlation coefficient was 0.57 for the conventional method, with coefficients ranging from 0.47 to 0.81 for each image combination method. The MAE was 0.44 L for the conventional method, with MAEs ranging from 0.29 to 0.38 L for each radiomic feature combination. Figure 2a,b illustrates the relationship between the predicted and measured FEV_1_ values for the conventional method and radiomic feature combination no. 4. This combination demonstrated the highest correlation and lowest MAE between the measured and predicted FEV_1_ values.

**FIGURE 3:**
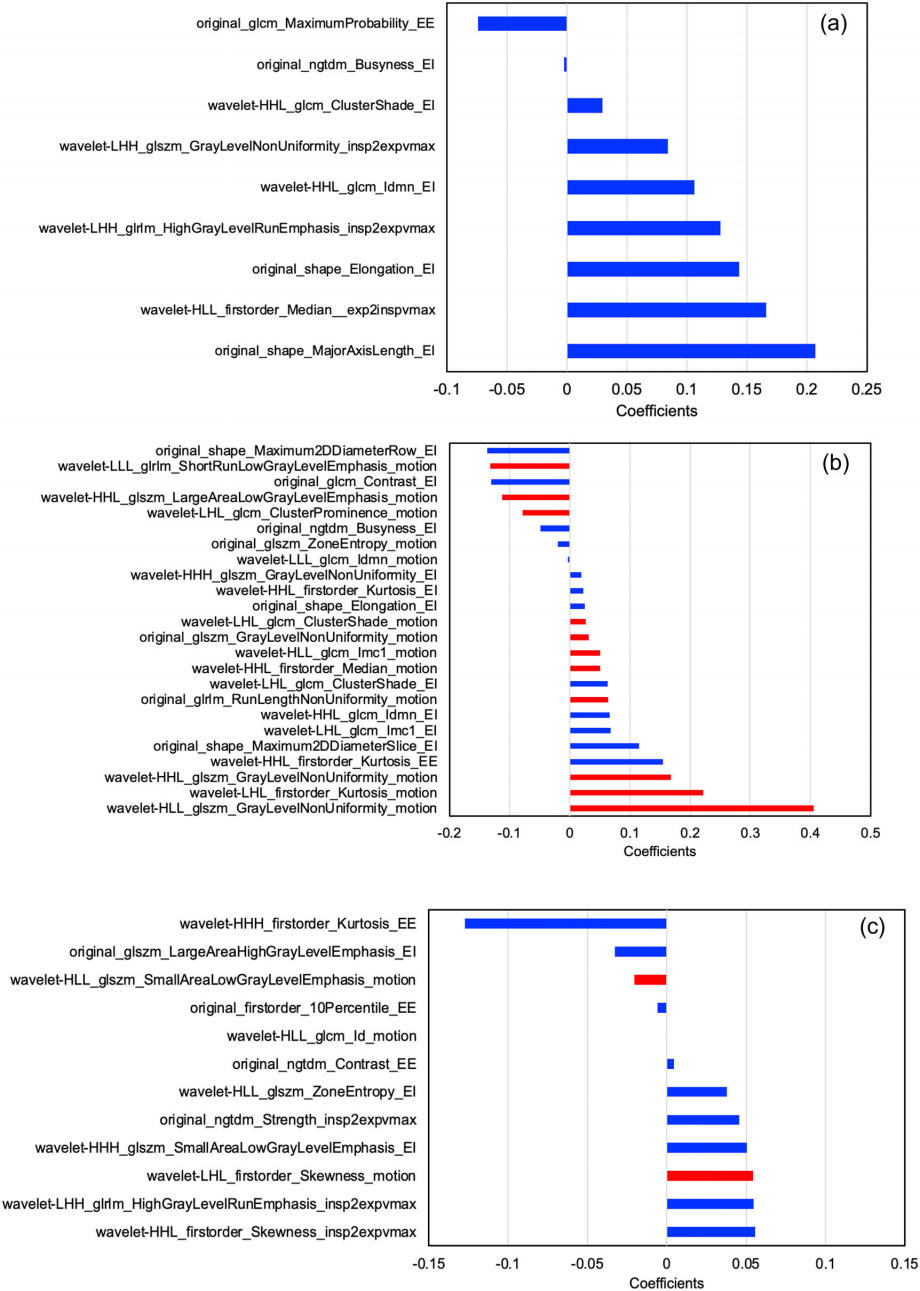
Selected radiomic features used to construct the prediction models via LASSO regression for (a) FEV_1_ using radiomic feature combination no. 4 and (b) FVC using radiomic feature combination no. 5, and (c) classification of FEV_1_/FVC > 70% using radiomic feature combination no. 6. Red indicates respiratory motion map‐based radiomic features. Feature names follow the convention [phase]_[feature], where EI = end‐inspiration, EE = end‐expiration, insp2exp_vmax_ = the respiratory phase at which the lung area shows the maximum rate of decrease from inspiration to expiration (i.e., the phase of maximum expiratory flow; “2” denotes “to”), and exp2insp_vmax_ = the respiratory phase at which the lung area shows the maximum rate of increase from expiration to inspiration (i.e., the phase of maximum inspiratory flow). RM = respiratory motion map. Wavelet‐derived features are indicated by the prefix “wavelet‐”, reflecting spatial frequency characteristics after wavelet decomposition.

Figure 3a displays the selected radiomic features used to construct the predictive model for FEV_1_. Specifically, the selected features spanned all four DCR phases —EI, EE, insp2exp_vmax_, and exp2insp_vmax_.

Table 5 presents the Pearson correlation coefficient and MAE for each prediction model in relation to FVC. For the training cohort, the Pearson correlation coefficient was 0.72 for the conventional method, with coefficients ranging from 0.93 to 0.98 for each radiomic feature combination. The MAE was 0.47 L for the conventional method, with MAEs ranging from 0.16 L to 0.27 L for each radiomic feature combination.

**TABLE 5:** The Pearson correlation coefficient and MAE for each FVC predictive model.

	Train cohort		Test cohort	
No.	R	MAE	R	MAE
conventional	0.72	0.47	0.48	0.50
1	0.93	0.27	0.75	0.37
2	0.97	0.23	0.49	0.51
3	0.98	0.18	0.68	0.42
4	0.94	0.24	0.69	0.38
5	0.98	0.18	0.80	0.34
6	0.98	0.16	0.78	0.35

Abbreviations: MAE, mean absolute error; R, Pearson correlation coefficient.

For the test cohort, the Pearson correlation coefficient was 0.48 for the conventional method, with coefficients ranging from 0.49 to 0.80 for each image combination method. The MAE was 0.50 L for the conventional method, with MAEs ranging from 0.34 L to 0.51 L for each radiomic feature combination. Figure 2c,d illustrates the relationship between the predicted and measured FVC values for the conventional method and image combination no. 5. This combination exhibited the highest correlation and lowest MAE between the actual and predicted FVC values.

Figure 3b displays the selected radiomic features used to construct the predictive model for FVC. Several radiomic features of the respiratory motion map were selected.

### 3.2 Classification model of FEV_1_/FVC over 70%

Table 6 presents the AUC, accuracy, sensitivity, specificity, PPV, and NPV of the classification model with FEV_1_/FVC > 70% for the conventional method and for each radiomic feature combination. In the conventional method, AUC for classification of FEV_1_/FVC > 70% was 0.68 (accuracy: 0.67, sensitivity: 0.73, specificity: 0.61, PPV: 0.70, NPV: 0.64) in the training cohort. For the test cohort, the AUC was 0.71 (accuracy: 0.68, sensitivity: 0.63, specificity: 0.74, PPV: 0.71, NPV: 0.67).

**TABLE 6:** The AUC, accuracy, sensitivity, specificity, PPV, and NPV for each radiomic feature combination.

	Train cohort						Test cohort					
No.	AUC	Accuracy	Sensitivity	Specificity	PPV	NPV	AUC	Accuracy	Sensitivity	Specificity	PPV	NPV
Conventional	0.68	0.67	0.73	0.61	0.7	0.64	0.71	0.68	0.63	0.74	0.71	0.67
1	0.74	0.67	0.71	0.63	0.71	0.63	0.78	0.82	0.79	0.84	0.83	0.80
2	0.82	0.78	0.77	0.79	0.82	0.73	0.65	0.63	0.63	0.63	0.63	0.63
3	0.85	0.77	0.75	0.79	0.82	0.71	0.71	0.66	0.63	0.68	0.67	0.65
4	0.86	0.78	0.81	0.74	0.8	0.76	0.78	0.74	0.79	0.68	0.71	0.76
5	0.89	0.8	0.79	0.82	0.84	0.76	0.76	0.71	0.74	0.68	0.7	0.72
6	0.89	0.83	0.83	0.82	0.85	0.79	0.77	0.76	0.79	0.74	0.75	0.78

Abbreviations: AUC, area under the curve; NPV, negative predictive value; PPV, positive predictive value.

The performance of the classification model of radiomic feature combination no. 6 for the training and test cohorts was well balanced.

Figure 3c displays the selected radiomic features used to construct the classification model of FEV_1_/FVC > 70%. Selected features included radiomic descriptors from DCR at EI, EE, and insp2exp_vmax_, together with respiratory motion map (RM) features (red bars).

### 3.3 Robustness of selected radiomic features

**FIGURE 4:**
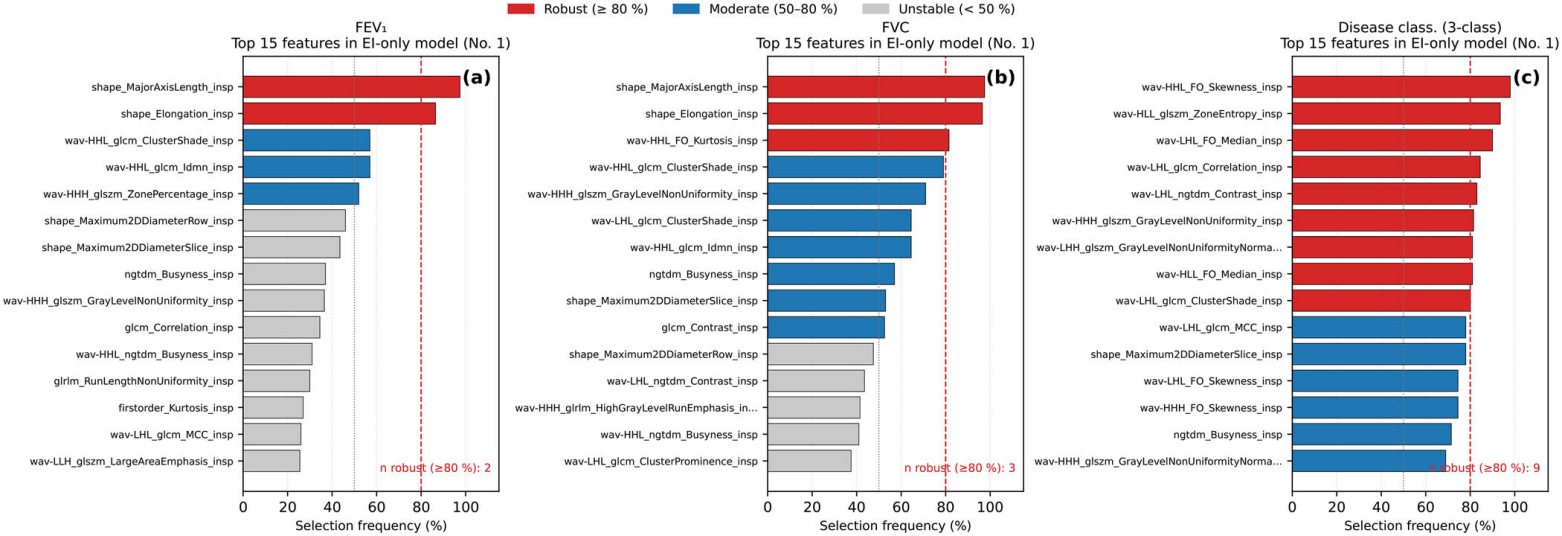
Bootstrap stability analysis of radiomic features for the three principal clinical targets, showing the top 15 features by selection frequency over 200 bootstrap resamples in the inspiration‐only (no. 1) feature combination. (a) FEV_1_: inspiratory shape descriptors (major‐axis length and elongation) emerge as the most stable, with two features above the 80 % robustness threshold. (b) FVC: the same two inspiratory shape descriptors together with additional shape features, three robust features in total. (c) Three‐class disease classification (asthma vs. COPD vs. interstitial pneumonia): wavelet‐derived texture features dominate (nine robust features), distinctly different from the regression signature. Red bars: features with ≥ 80 % selection frequency (robust); blue bars: 50%–80 %; grey bars: < 50 %. Red dashed line: 80 % robustness cut‐off.

Bootstrap stability selection (200 resamples) identified a small number of consistently selected (≥ 80 % selection frequency) radiomic features per target. For FEV_1_ prediction, the two most stable features across all inspiration‐containing combinations were original_shape_MajorAxisLength_EI (97.5 %) and original_shape_Elongation_EI (86.5 %). For FVC prediction, the same two features were selected in 97.5 % and 96.5 % of bootstrap resamples, respectively, together with original_shape_MajorAxisLength_EE (96.0 % in the expiration‐only combination no. 2). When the motion map was added to the feature pool, the wavelet‐derived wavelet‐HHL_glszm_GrayLevelNonUniformity_RM was selected in 82–89 % of resamples for FVC but did not reach the 80 % threshold for FEV_1_. Per‐combination robust‐feature counts and full feature lists are provided in Tables S1 and S2 and Figure 4.

### 3.4 Universal minimal‐feature predictive model

**FIGURE 5:**
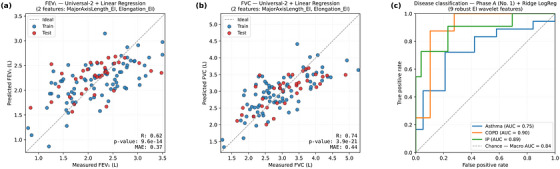
Predictive performance of the universal minimal and robust‐feature models. (a) FEV_1_ prediction using only two universally robust inspiration shape features (MajorAxisLength_EI and Elongation_EI) and ordinary linear regression on a representative 70: 30 train–test split (seed = 42): *R* = 0.62, *p* < 0.001, MAE = 0.37 L. (b) FVC prediction using the same two features: *R* = 0.74, *p* < 0.001, MAE = 0.44 L. (c) ROC curves for three‐class disease classification (asthma vs. COPD vs. interstitial pneumonia) using the nine bootstrap‐robust inspiration wavelet‐texture features and ridge multinomial logistic regression: per‐class one‐versus‐rest AUCs of 0.75 (asthma), 0.90 (COPD), and 0.89 (IP); macro AUC = 0.84. Blue circles: training‐cohort samples; red circles: test‐cohort samples; grey dashed line: ideal (*y* = *x* for scatter; chance line for ROC).

A parsimonious “Universal Minimal” model using only the two universally robust inspiration shape descriptors (MajorAxisLength_EI and Elongation_EI) achieved a mean test Pearson R of 0.61 (95 % CI 0.38–0.76) for FEV_1_ and 0.74 (95 % CI 0.61–0.86) for FVC over 50 random 70: 30 splits, with mean test MAE of 0.38 L and 0.44 L, respectively. With these two features, ordinary linear regression matched ridge and LASSO regression and outperformed random forest (mean test *R* = 0.61 vs. 0.54 for FEV_1_ and 0.74 vs. 0.71 for FVC; Table S4). The single‐split predicted‐versus‐measured scatter plots are shown in Figure 5a,b. Notably, the Universal Minimal + Linear model approached the performance of the published combination no. 5 for FVC (*R* = 0.80 in the single‐split primary analysis, mean *R* = 0.74 in the multi‐seed analysis) using only 2 features instead of the approximately 31 features selected by the full feature‐selection pipeline for Combination 5.

### 3.5 Multi‐class classification of respiratory disease

Of 122 patients with a definitive single‐disease diagnosis (asthma, *n* = 59; COPD, *n* = 27; IP, *n* = 36), bootstrap stability identified 4–9 robust radiomic features per combination, with wavelet‐HHL_firstorder_Skewness_EI (98.0 %) being the most consistently selected. Notably, the universally robust features for FEV_1_/FVC regression (shape descriptors) were not consistently selected for disease classification, which instead favoured wavelet‐derived texture features. Using only the nine robust features from the EI‐only combination (no. 1) and L2‐penalised multinomial logistic regression, we achieved a mean macro‐averaged one‐vs‐rest ROC AUC of 0.81 (95 % CI 0.74–0.91) over 50 stratified random splits (Table S5). For a representative single split (seed = 42), per‐class one‐vs‐rest AUCs were 0.75 for asthma, 0.90 for COPD, and 0.89 for IP, with a macro AUC of 0.84 (Figure 5c). Random‐forest classification on the same robust features yielded a comparable mean macro AUC of 0.79. To benchmark this against the clinical reference standard, we repeated the identical three‐class classification using spirometry under the same 50‐split framework: measured FEV_1_ and FVC alone gave mean macro AUCs of 0.54 and 0.61, respectively, and 0.74 in combination — all significantly below the DCR radiomic model (0.81; paired Wilcoxon signed‐rank *p* < 0.001; Figure S4, Table S6). DCR radiomics therefore, matched or exceeded the disease‐classification performance of measured FEV_1_ and FVC.

## 4 DISCUSSION

In this study, we constructed a prediction model for PFT using DCR in four respiratory phases and a respiratory motion map. The main findings indicated that DCR at each respiratory phase and respiratory motion map‐based radiomic features could predict FEV_1_ and FVC and classify FEV_1_/FVC > 70%. In the primary 70:30 split, combination no. 4 (EI + EE + insp2expvmax + exp2inspvmax) yielded the best test‐cohort performance for FEV_1_ prediction, and combination no. 5 (EI + EE + RM) yielded the best test‐cohort performance for FVC prediction. These single‐split estimates are further contextualised by the multi‐seed sensitivity analysis discussed below. Moreover, our findings demonstrated that the AUC for FEV_1_/FVC on DCR and respiratory motion map‐based radiomic feature combination no. 6 (EI + EE + insp2exp_vmax_ + exp2insp_vmax_ + RM) was higher than that of the conventional model.

Although many studies have clinically evaluated DCR for perfusion,^27^, ^28^ specific clinical evaluation of DCR for quantitative pulmonary ventilation prediction has remained limited. Several studies have investigated the relationship between dynamic lung area changes on DCR and ventilatory function indices, including projected lung area analysis in patients with COPD and healthy subjects,^29^ DCR assessment in chronic obstructive or restrictive lung disease,^30^ FVC prediction using DCR in interstitial lung disease,^31^ lung volume calculation using DCR in patients with cystic fibrosis,^32^ and FVC correlation with biphasic DCR.^33^ However, none of these studies have directly predicted quantitative PFT values from radiomic features incorporating respiratory motion maps, which capture regional heterogeneity of lung parenchymal dynamics. This is due to the complexity of pulmonary function, which cannot be straightforwardly expressed in terms of pixel values in the lung parenchyma.^10^ To the best of our knowledge, this study proposes a novel approach based on DCR and respiratory motion map‐based radiomic features and represents the first attempt to estimate PFT results from radiomic features directly extracted from DCR images and respiratory motion maps.

Although DCR in this study was performed during guided deep breathing rather than forced expiratory maneuvers, the radiomic features extracted from DCR images and respiratory motion maps capture structural and mechanical properties of the lung parenchyma — including regional ventilation heterogeneity, parenchymal compliance, and diaphragmatic excursion — that are themselves determinants of forced expiratory performance. Patients with airflow limitation (low FEV_1_/FVC) exhibit characteristic patterns of regional air trapping, reduced parenchymal displacement, and altered diaphragmatic mechanics that are detectable even during non‐forced breathing. Hino et al.^15^ demonstrated that lung parenchymal motion vectors during DCR differ significantly between COPD patients and healthy controls. Thus, while DCR does not directly measure forced expiratory flow, it can serve as an indirect surrogate by capturing the underlying structural‐functional substrate that determines spirometric performance.

It should be noted that the respiratory motion map employed in this study was computed in an Eulerian (image‐space) framework: optical flow magnitudes were accumulated at each fixed pixel location rather than tracking individual anatomical structures across frames in a Lagrangian sense. Although this approach does not explicitly follow tissue‐element trajectories, the resulting scalar field captures the spatial distribution of cumulative motion at each anatomical location, providing a physiologically meaningful representation of regional ventilation heterogeneity. Eulerian motion representations have been widely adopted in DCR research, including vector‐field analyses of lung parenchymal dynamics, and have demonstrated the ability to discriminate between healthy and diseased lungs. Future studies may benefit from incorporating Lagrangian tracking or deformable registration techniques to complement the Eulerian motion map with explicit tissue‐trajectory information.

Hino et al.^15^ assessed the difference in lung motion using the vector field of DCR with optical flow in healthy volunteers and patients with COPD and reported that the lung parenchyma moved faster in patients with COPD than in healthy controls.^15^ Therefore, in this study, radiomic features derived from DCRs at the respiratory phases of maximum lung area variation (insp2exp_vmax_, exp2insp_vmax_), along with EE, EI, and respiratory motion maps calculated using optical flow, were used to construct the prediction model. For the prediction of FEV_1_, FVC, and classification of FEV_1_/FVC > 70%, the incorporation of radiomic features derived from the DCRs at EE, insp2exp_vmax_, exp2insp_vmax_, and EI along with radiomic features derived from respiratory motion maps improved the accuracy of the predictive model.

Park et al.^8^ developed a prediction model for PFTs using volumetric CT images and deep learning techniques. In their independent test cohort, they achieved an MAE of 0.22 L and correlation coefficients of 0.94 for FVC and 0.91 for FEV_1_. Furthermore, the AUC was 0.85 for classifying FEV_1_/FVC > 70%. The DCRs consisted of planar data rather than volumetric data. Radiomic features extracted from CT images may potentially provide more accurate reflections of lung function compared to those from DCR. However, radiomic features are often criticised for being low‐level and less abstract than deep features, which may limit their informativeness and degrade model performance. Therefore, in our test cohort, our model did not achieve the same level of accuracy as theirs.

Ieko et al.^9^ estimated PFT using inspiratory CT‐radiomic features based on machine learning. Although their model was not validated in an independent test cohort, the results of our proposed model were comparable to theirs. Their model employed only the original radiomic features. In a previous study, Prinzi et al.^34^ investigated the impact of wavelet filters on the predictive ability of chest x‐ray images. Despite the trade‐off between interpretability and performance, wavelet‐derived radiomic features offer a pathway to build robust predictive models.

Our predictive model demonstrated a more accurate prediction of FEV_1_ and FVC than did the conventional clinical models. Additionally, it was able to effectively classify FEV_1_/FVC > 70%. Kristensen et al.^4^ developed a prediction model for PFT using body measurements and demographic data based on a random forest machine learning algorithm. However, their model achieved highly accurate predictions of FEV_1_ and FVC because the prediction model was built based on data from subjects with relatively preserved pulmonary function, in whom FEV_1_/FVC was consistently above 70%.

Ueyama et al.^31^ demonstrated that a simple multiple linear regression model using only projected lung areas at maximum inspiration and expiration (EI + EE) achieved an MAE of 0.27 L for FVC prediction in patients with interstitial lung disease. Our radiomic feature combination no. 5 (EI + EE + RM) achieved an MAE of 0.34 L in the test cohort. Although slightly higher than the MAE reported by Ueyama et al., this difference should be interpreted with caution given the differences in patient populations: our study included a heterogeneous mix of respiratory diseases including asthma, COPD, IP, and ACOS, whereas Ueyama et al. focused specifically on patients with interstitial lung disease. The respiratory motion map (RM) captures regional heterogeneity of lung parenchymal motion that is not reflected in global lung area measurements alone, and this additional information may be particularly valuable in patients with spatially heterogeneous diseases such as COPD or IP. However, the RM requires additional computational processing, including optical flow calculation across DCR frames, which increases the complexity of the clinical workflow. Clinically, the simpler EI + EE model may be sufficient in resource‐limited settings or when rapid assessment is required, whereas the EI + EE + RM model may offer advantages when capturing regional ventilation heterogeneity is clinically relevant. The selection of an appropriate model should therefore be guided by the clinical context and the required level of prediction accuracy.

Bootstrap stability selection identified two inspiratory lung‐shape descriptors — major‐axis length and elongation of the inspiratory lung field — as the most reproducible DCR‐radiomic predictors of FEV_1_ and FVC, with selection frequencies of ≥ 86 % across 200 bootstrap resamples (Figure 4a,b). These two features are physiologically interpretable as surrogates of inspiratory lung volume and lung‐shape change during inspiration, respectively. Both are known to be reduced in obstructive and restrictive pulmonary disease, providing a mechanistic basis for their predictive value: a smaller inspiratory lung dimension reflects diminished inspiratory capacity, while reduced elongation reflects impaired expansion and altered compliance.

Building on this stability analysis, we constructed a parsimonious “Universal Minimal” model using only these two shape descriptors. Across 50 random 70: 30 splits, this two‐feature linear‐regression model achieved mean test Pearson R values of 0.61 for FEV_1_ and 0.74 for FVC — values comparable to the more complex feature combinations evaluated under the same multi‐seed framework (in which Combination 4 attained mean *R* = 0.47 for FEV_1_ and Combination 5 attained mean *R* = 0.74 for FVC), despite using only two features instead of approximately 13–31. With only two features, ordinary linear regression matched ridge and LASSO regression and outperformed the random‐forest classifier, consistent with the expectation that simpler learners are favoured when the number of predictors is small relative to the sample size. The clinical implication is that a highly interpretable two‐feature model — derivable directly from automated lung segmentation on DCR — may suffice for first‐pass pulmonary‐function estimation, with potential for transparent quality control in clinical deployment.

Beyond the prediction of continuous spirometric values, we also evaluated whether DCR radiomic features can directly classify the underlying respiratory disease. In 122 patients with a definitive single‐disease diagnosis (asthma, *n* = 59; COPD, *n* = 27; IP, *n* = 36), bootstrap‐robust wavelet‐texture features from the inspiration phase, combined with a multinomial ridge logistic‐regression classifier, yielded a mean macro‐averaged one‐vs‐rest ROC AUC of 0.81 (95 % CI 0.74–0.91), with per‐class AUCs of 0.75 for asthma, 0.90 for COPD, and 0.89 for IP (Figure 5c). This demonstrates that DCR‐derived radiomic features carry directly classifiable disease‐specific signals, supporting the suggestion that DCR may be well suited for direct disease classification in addition to spirometric prediction.

An important insight emerging from these complementary analyses is that different clinical prediction tasks favour different radiomic feature signatures. For absolute pulmonary volume prediction (FEV_1_ and FVC), the dominant robust features were inspiratory lung‐shape descriptors that directly index lung expansion. For disease classification, the dominant robust features were wavelet‐derived texture descriptors that capture the heterogeneity of lung parenchyma — a property modulated by the structural changes of emphysema in COPD and fibrotic distortion in interstitial pneumonia. Indeed, the Universal Minimal shape features that excelled at FEV_1_/FVC regression were not consistently selected for disease classification. This task‐specific divergence is consistent with the underlying physiology — overall volume reflects gross lung mechanics, whereas texture reflects parenchymal microstructure — and reinforces the need to identify the relevant feature signature for each clinical question rather than relying on a single universal radiomic profile.

One limitation of this study is the small sample size. Therefore, our results must be verified using larger sample sizes and multicentre data. Additionally, the predictive model constructed in this study was validated solely using data from our institution and was not tested using external data. Hence, future studies should include external datasets for validation purposes.

Furthermore, our cohort did not include healthy volunteer data. By adding data from healthy volunteers, the predictive regression and classification models could be improved.

Although we have characterised the stability of our radiomic feature selection to perturbations of the training sample by bootstrap resampling, a complementary assessment of stability to image‐acquisition perturbations would require dedicated test–retest imaging data, which were not available in this retrospective study. Quantifying intra‐scanner and inter‐vendor reproducibility of the proposed Universal Minimal shape features is therefore an important direction for future work. In addition, the disease‐classification analysis was performed on 122 patients with a definitive single‐disease diagnosis; patients with asthma–COPD overlap syndrome (*n* = 2) were excluded, and external multi‐centre validation will be required before clinical deployment.

Importantly, DCR was performed during guided deep breathing rather than forced expiratory maneuvers, and the radiomic features therefore reflect structural and mechanical correlates of forced expiratory function rather than the forced expiratory maneuver itself. The clinical role of DCR‐based pulmonary function estimation should therefore be conceived as a complementary or surrogate assessment, particularly in settings where spirometry is unreliable, contraindicated, or unavailable, rather than as a replacement for direct spirometric measurement.

Additionally, radiomic features in this study were extracted from for‐presentation (post‐processed) images rather than for‐processing (raw) images. For‐presentation images undergo non‐linear transformations such as gradation curve adjustment and edge enhancement, which may affect the reproducibility and quantitative accuracy of radiomic features. Future studies should evaluate the impact of image processing pipelines on radiomic feature stability and consider using for‐processing images to maximise quantitative fidelity.

Furthermore, the respiratory motion map was computed in an Eulerian framework, accumulating optical flow magnitudes at fixed pixel locations rather than tracking individual tissue elements across frames. While this approach provides a clinically useful representation of regional motion heterogeneity, it does not account for through‐plane motion or anatomical correspondence between frames. Lagrangian tracking or deformable image registration approaches may provide complementary information and should be explored in future work.

## 5 CONCLUSION

In this single‐center retrospective feasibility study, bootstrap stability selection identified two universally robust inspiratory lung‐shape features (major‐axis length and elongation) that, when used in a simple two‐feature linear regression, attained mean test Pearson R values of 0.61 for FEV_1_ and 0.74 for FVC over 50 random 70:30 splits, outperforming conventional demographic‐based prediction. A separate wavelet‐texture signature supported direct three‐class classification of asthma, COPD, and interstitial pneumonia with a mean macro‐averaged AUC of 0.81, matching or exceeding classification based on measured FEV_1_ and FVC (Figure S4). These findings indicate that DCR radiomics may serve as a parsimonious, interpretable, and reproducible complementary assessment of lung function, particularly in patients for whom spirometry is unreliable or infeasible. External validation in larger, multi‐center cohorts is required before clinical translation.

## AUTHOR CONTRIBUTIONS

Takehiro Shiinoki and Yuki Yuasa carried out the experiment. Takehiro Shiinoki wrote the manuscript with support from Yuki Yuasa, Tsunahiko Hirano, Maki Asami‐Noyama, Kazuto Matsunaga, and Hidekazu Tanaka supplied available data in terms of this study and discussed. All authors discussed the results and contributed to the final manuscript.

## CONFLICT OF INTEREST STATEMENT

The authors declare no conflicts of interest.

## ETHICS STATEMENT

This single‐centre retrospective study was approved by the Institutional Review Board of Yamaguchi University Hospital (approval no. 2024–081). Written informed consent was waived because of the retrospective design. This retrospective study was performed according to the relevant institutional guidelines and regulations.

## GENERATIVE AI STATEMENT

The authors declare that ChatGPT was used in the drafting of this manuscript. Specifically, ChatGPT was used for language improvement. After using this tool, the authors reviewed and edited the content to ensure its accuracy and compliance with academic standards. The responsibility for the content of this paper rests entirely with the authors.

## Supporting information



Supporting Information

## Data Availability

The data that support the findings of this study are available from the corresponding author upon reasonable request, subject to institutional review board approval and data sharing agreements.
