# Supplementary material for: Estimation of pulmonary function from time‐resolved dynamic chest radiography using machine learning in patients with respiratory disease
Source: J Appl Clin Med Phys. 2026 Jul 27;27(8):e70717. doi: 10.1002/acm2.70717 (PMC13404249; doi:10.1002/acm2.70717)
Supplement: Supplementary file 1 — Supporting Information [file ACM2-27-e70717-s001.zip › 2026-09352-sup-0002--S.docx]

**Supplement 1**

**Data acquisition protocol for dynamic chest x-ray images**

sFigure 1 shows the data acquisition protocol for dynamic chest radiography (DCR). The protocol instructed patients to inhale deeply for five seconds, exhale for five seconds, and hold their breath for two seconds, following automated instructions. The total time of radiation exposure was 20 seconds, yielding 300 frames for analysis. However, the acquisition of DCR data in accordance with the protocol may vary depending on the radiographer's imaging technique and the patient's understanding of the imaging procedure.

**
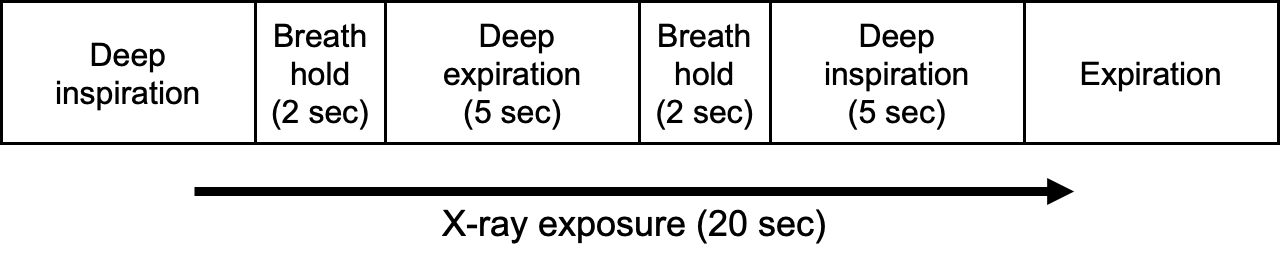
**

**sFigure 1.** Data acquisition protocol for dynamic chest x-ray images

**Definition of the respiratory phase of sequential dynamic chest x-ray images**

**
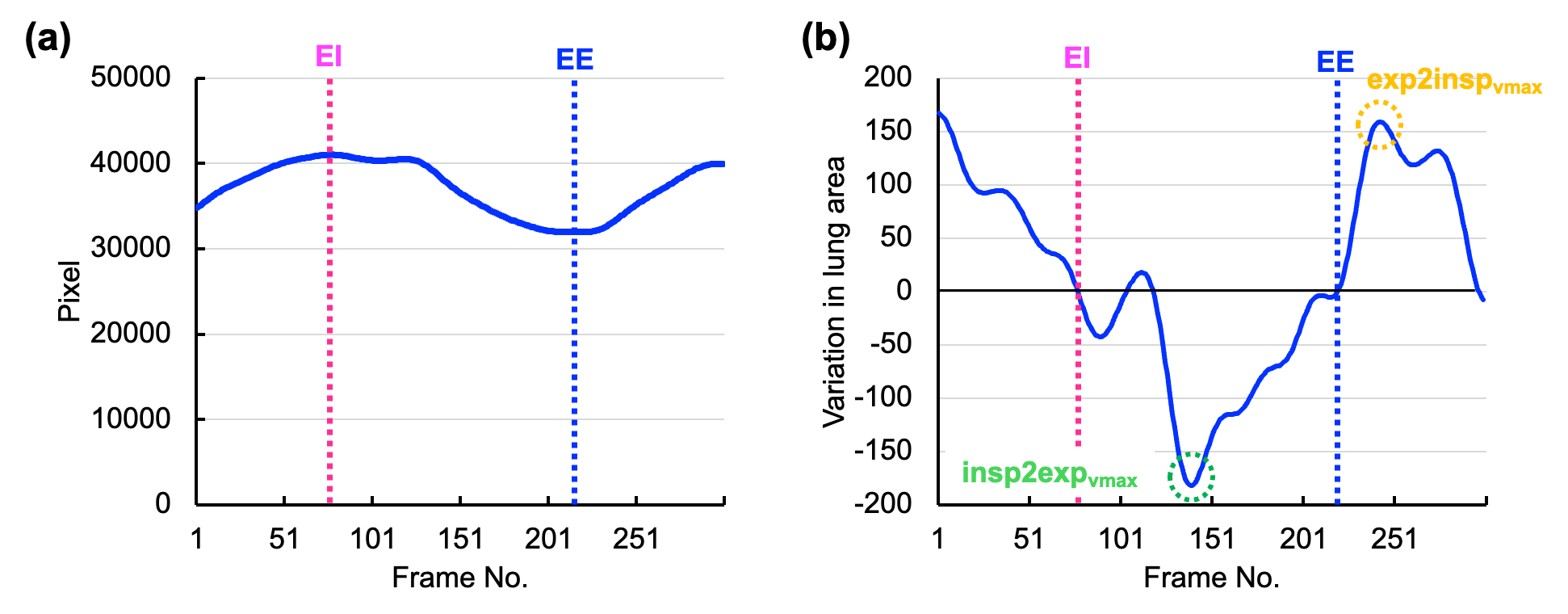
**

**sFigure 2** **(a)** Lung area relative to frame number; **(b)** Variation in lung area relative to frame number

sFigure 2 (a) shows the lung area relative to frame number. The DCR frame with the maximum lung area was labelled as end-inspiration (EI). The DCR frame with the minimum lung area was labelled as end-expiration (EE). sFigure 2 (b) shows the variation in the lung area relative to frame number. The variation in lung area was calculated by differentiating the lung area. The DCR frame with the minimum lung area variation was labelled as insp2expvmax. The DCR frame with the maximum lung area variation was labelled as exp2inspvmax. Only frames occurring after the DCR frame at end-inspiration (EI) were analysed.

**Supplement 2**

**Bootstrap stability selection, multi-algorithm sensitivity, and multi-class disease classification**

**S3.1 Methods**

Bootstrap stability selection. The complete three-stage feature-selection pipeline of the primary analysis — Spearman redundancy filter at |ρ| > 0.80 → Pearson-correlation-with-target prefilter at |r| > 0.20 for regression targets (FEV₁ and FVC), or ANOVA F-test prefilter at p < 0.05 for the multi-class disease-classification target → LASSO regression (for regression) or L1-penalised multinomial logistic regression (for classification), with internal 10-fold cross-validation for the penalty parameter — was applied to 200 bootstrap resamples of the study cohort. For every radiomic-feature combination and every clinical target, the selection frequency of each feature was computed as the proportion of bootstrap iterations in which it was retained with a non-zero coefficient. Features with selection frequency ≥ 0.80 were defined a priori as 'robust' (Meinshausen and Bühlmann, J. R. Stat. Soc. B 2010).

Multi-seed multi-algorithm sensitivity analysis. For each of the six published radiomic-feature combinations, the predictive performance was re-evaluated over 50 independent random 70 : 30 train–test splits. Within each split, the feature-selection pipeline was applied to the training fold only to prevent test-data leakage, and four regression algorithms were compared on the resulting feature subset: ordinary linear regression, ridge regression (with cross-validated L2 penalty), LASSO regression (with cross-validated L1 penalty), and random forest with 200 trees, min-samples-leaf = 2. For each (combination, algorithm) pair the test-cohort Pearson R and mean absolute error were aggregated as mean and 95 % percentile confidence interval across the 50 splits. A parsimonious 'Universal Minimal' feature model — using only the two (or, in a sensitivity variant, three) universally robust inspiration shape features identified by the bootstrap analysis (original_shape_MajorAxisLength_EI, original_shape_Elongation_EI, and optionally original_shape_MajorAxisLength_EE) — was evaluated identically. In the supplementary tables, the 2-feature and 3-feature variants are denoted 'B_Universal-2' and 'B_Universal-3', respectively.

Multi-class disease classification. Of the 124 patients in the analytic cohort, 122 had a definitive single-disease diagnosis recorded (asthma, n = 59; chronic obstructive pulmonary disease (COPD), n = 27; interstitial pneumonia (IP), n = 36); two patients with asthma–COPD overlap syndrome were excluded. Four classification algorithms — unpenalised multinomial logistic regression, L2-penalised (ridge) and L1-penalised (LASSO) multinomial logistic regression with cross-validated regularisation strength, and a 200-tree random forest classifier — were compared over 50 stratified random 70 : 30 train–test splits, with class_weight = 'balanced' to mitigate class imbalance. Performance was reported as accuracy, macro-averaged F1 score, and macro-averaged one-vs-rest ROC AUC.

**S3.2 Results**

Robustness of radiomic features. Bootstrap stability selection identified a small number of consistently selected (≥ 80 % frequency) radiomic features per clinical target (Figure 4, sTable 1). For FEV₁ and FVC regression, the same two inspiratory shape descriptors emerged as universally robust across all inspiration-containing feature combinations: original_shape_MajorAxisLength_EI (97.5 % for both FEV₁ and FVC in the EI-only model) and original_shape_Elongation_EI (86.5 % for FEV₁, 96.5 % for FVC). One additional expiration shape descriptor (original_shape_MajorAxisLength_EE) was robust for FVC only, and one wavelet-derived motion-map feature (wavelet-HHL_glszm_GrayLevelNonUniformity_RM) was robust for FVC in motion-inclusive combinations (sTable 2). For the disease-classification target the robust signature was substantially different and dominated by wavelet-derived texture features such as wavelet-HHL_firstorder_Skewness_EI (98.0 %), wavelet-HLL_glszm_ZoneEntropy_EE (100.0 %), and wavelet-LHL_gldm_DependenceNonUniformityNormalized_RM (99.5 % in motion-inclusive combinations) — see sTable 3.

Multi-seed multi-algorithm sensitivity. Across 50 random 70 : 30 splits, the Universal Minimal (2-feature) model attained mean test Pearson R values of 0.61 (95 % CI 0.38–0.76) for FEV₁ and 0.74 (95 % CI 0.61–0.86) for FVC, with mean test MAE of 0.38 L and 0.44 L, respectively (sTable 4). With only two features, ordinary linear regression, ridge regression, and LASSO performed identically and outperformed random forest, which loses its advantage when the number of predictors is very small. For the more complex full feature-selection pipeline (Phase 0), random forest provided the highest mean R for motion-inclusive combinations, but the gain over the Universal Minimal (2-feature) model was modest and accompanied by wider confidence intervals.

Multi-class disease classification. Using only the nine bootstrap-robust inspiration wavelet features from the EI-only combination (No. 1) together with a ridge multinomial logistic regression classifier, the cohort of 122 patients could be classified into asthma, COPD, and IP with a mean macro-averaged one-vs-rest ROC AUC of 0.81 (95 % CI 0.74–0.91) over 50 stratified splits (sTable 5 and Figure 5(c)). For a representative single split (seed = 42), per-class one-vs-rest AUCs were 0.75 for asthma, 0.90 for COPD, and 0.89 for IP, with a macro AUC of 0.84. Random-forest classification on the same robust features yielded a comparable mean macro AUC of 0.79. To benchmark these results against the clinical reference standard, the identical three-class classification was repeated using spirometry values as predictors under the same 50-split, four-algorithm framework. Measured FEV₁ and FVC alone yielded mean macro one-vs-rest AUCs of 0.54 and 0.61, respectively, and 0.74 in combination, each significantly below the DCR radiomic model (0.81; paired Wilcoxon signed-rank p < 0.001 for all comparisons; sFigure 4 and sTable 6). Even a comprehensive spirometry panel that additionally included the FEV₁/FVC ratio and the age/sex/height-adjusted percent-predicted values was only statistically equivalent to the DCR model on discrimination (mean macro AUC 0.83 versus 0.81; paired p = 0.10), and that panel encodes demographic information not available to the radiograph.

**S3.3 Supplementary Figure**

sFigure 3. Bootstrap stability and multi-algorithm prediction performance for FEV₁, FVC, and 3-class disease classification. Top row: feature selection frequency distributions over 200 bootstrap resamples for each of the six published feature combinations; red dashed line = 80 % robustness cut-off. Bottom row: mean test Pearson R (FEV₁, FVC) or macro AUC (disease) ± 95 % CI over 50 random 70 : 30 splits for each of the four algorithms compared in Phase 0 (full pipeline), Phase A (robust-only features), and the Universal Minimal (2- and 3-feature) models. In the figure, "P0" and "PA" denote Phase 0 and Phase A, respectively, and "U2" / "U3" denote the 2- and 3-feature Universal Minimal variants.


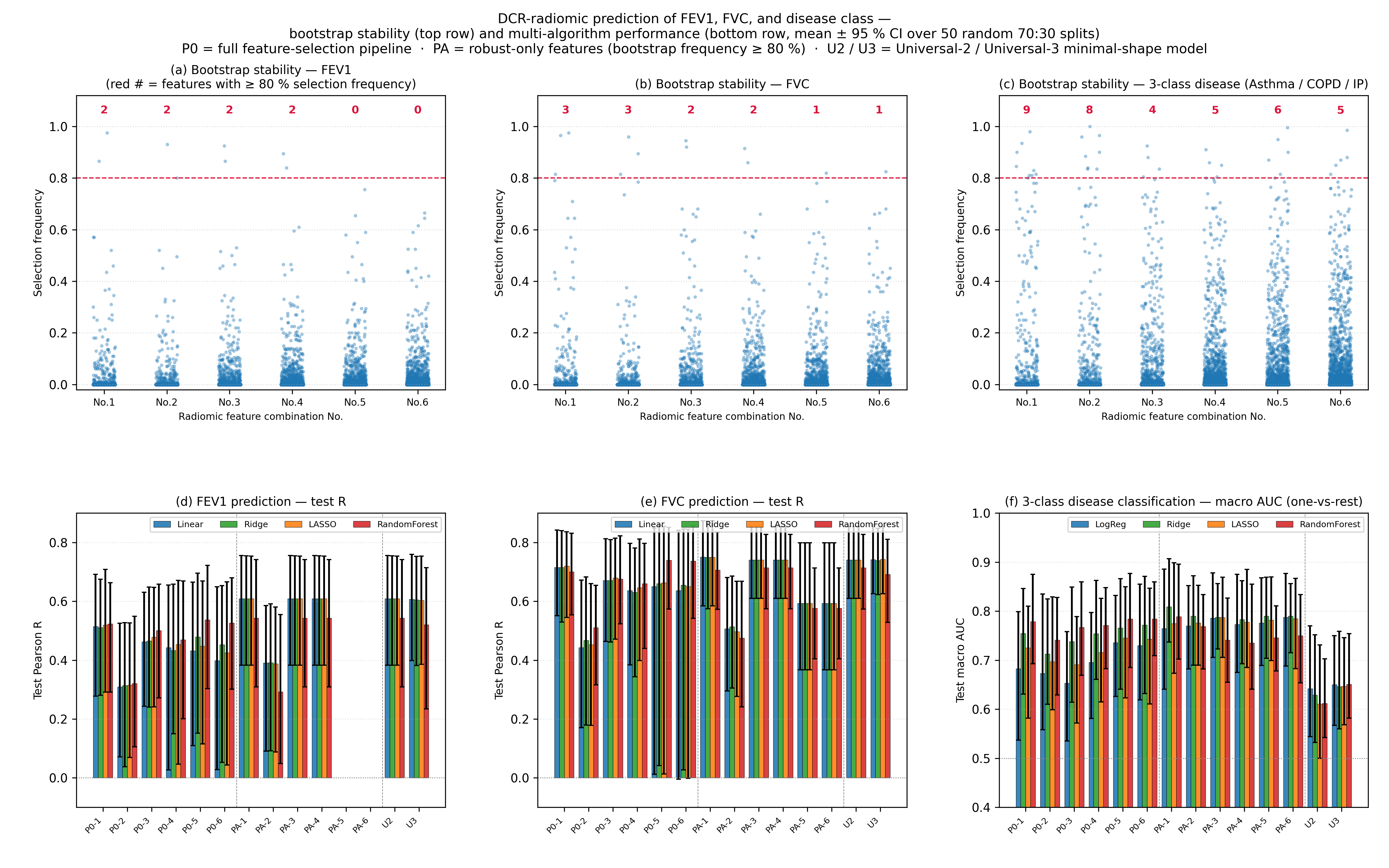


sFigure 4. Three-class disease classification (asthma vs COPD vs interstitial pneumonia) using DCR radiomics compared with spirometry (FEV₁ and FVC). (a–d) Per-class one-vs-rest ROC curves on the same representative 70 : 30 split as Figure 5(c) (seed = 42), using ridge multinomial logistic regression: (a) DCR radiomics with the nine bootstrap-robust end-inspiration wavelet-texture features (macro AUC = 0.84; identical to Figure 5(c)); (b) FEV₁ alone (macro AUC = 0.49); (c) FVC alone (macro AUC = 0.67); (d) FEV₁ + FVC (macro AUC = 0.74). (e) Macro one-vs-rest AUC (mean ± 95 % CI over 50 stratified 70 : 30 splits) for the DCR radiomic model (best robust-feature model, and an unbiased model with feature selection inside every fold) versus the spirometry models; the full spirometry panel (FEV₁, FVC, FEV₁/FVC, %FEV₁, %FVC) is shown in grey for reference. FEV₁ alone is close to chance (0.5) because both COPD (airflow obstruction) and interstitial pneumonia (restriction) lower FEV₁; measured FEV₁ and FVC together reach a macro AUC of 0.74, which remains below the DCR radiomic models (0.78–0.81; paired Wilcoxon signed-rank p < 0.001 for every comparison, DCR superior in 78–100 % of the 50 paired splits). All models were trained and evaluated on identical patients, splits, algorithm, scaling and metrics, so the comparison is paired seed-by-seed.


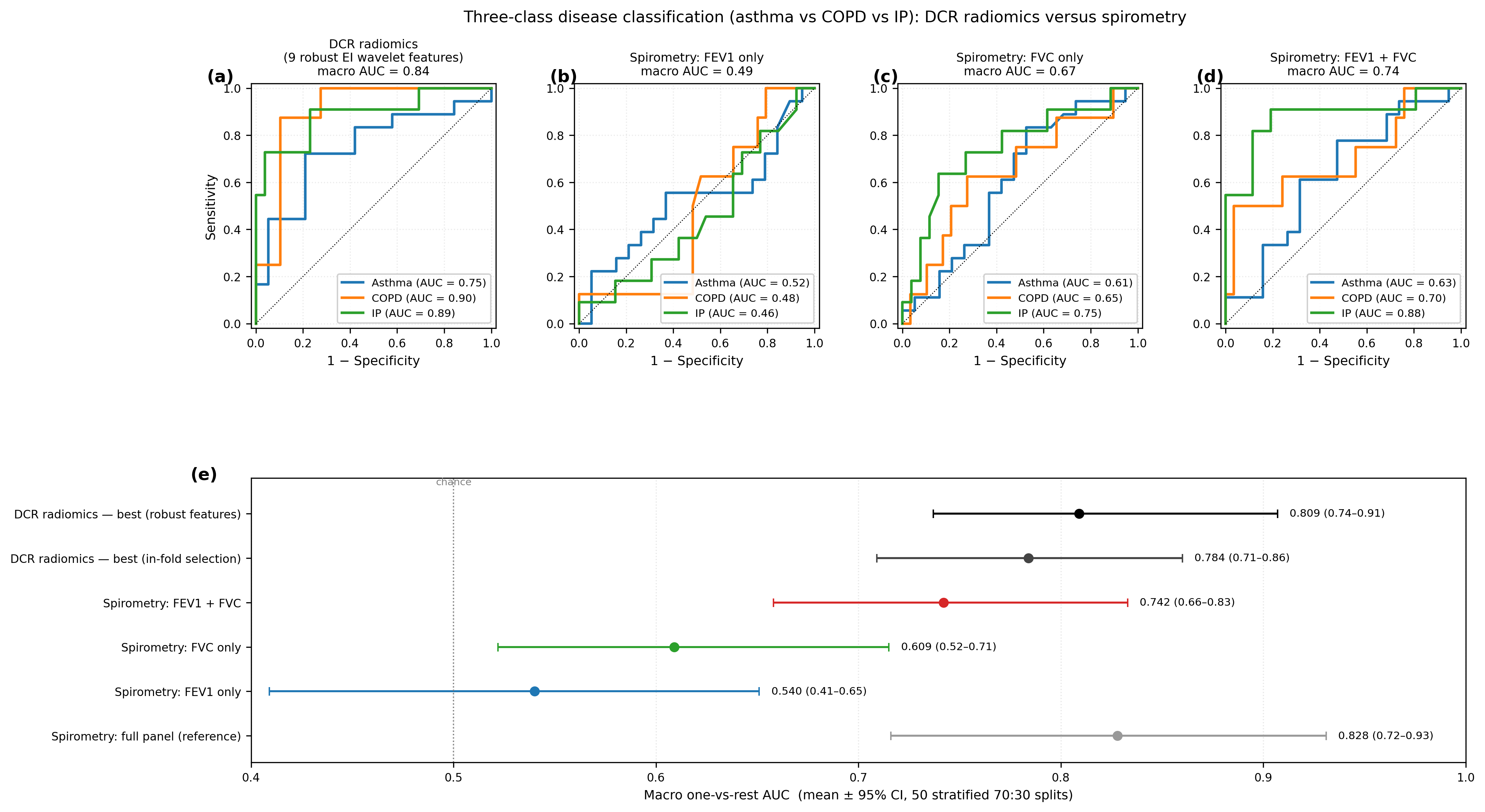


**S3.4 Supplementary Tables**

***sTable 1. Bootstrap stability summary for FEV₁, FVC, and 3-class disease classification.***

| **Target** | **Combination** | **n total features** | **n robust (≥80 %)** | **n robust (≥50 %)** | **Top-1 feature** | **Top-1 frequency** |
| --- | --- | --- | --- | --- | --- | --- |
| FEV₁ | 01_insp | 851 | 2 | 5 | original_shape_MajorAxisLength_insp | 0.975 |
| FEV₁ | 02_exp | 851 | 2 | 3 | original_shape_MajorAxisLength_exp | 0.930 |
| FEV₁ | 03_insp+exp | 1702 | 2 | 5 | original_shape_MajorAxisLength_insp | 0.925 |
| FEV₁ | 04_insp+inspmax | 1702 | 2 | 5 | original_shape_MajorAxisLength_insp | 0.965 |
| FEV₁ | 05_inspmax+exp | 1702 | 1 | 5 | original_shape_MajorAxisLength_insp_max_diff | 0.935 |
| FEV₁ | 06_exp+expmax | 1702 | 1 | 4 | original_shape_MajorAxisLength_exp | 0.810 |
| FEV₁ | 07_insp+inspmax+exp+expmax | 3404 | 2 | 4 | original_shape_MajorAxisLength_insp | 0.895 |
| FEV₁ | 08_insp+exp+motion | 2539 | 0 | 5 | wavelet-HHL_glszm_GrayLevelNonUniformity_motion | 0.755 |
| FEV₁ | 09_insp+inspmax+motion | 2539 | 0 | 7 | wavelet-HHL_glszm_GrayLevelNonUniformity_motion | 0.775 |
| FEV₁ | 10_inspmax+exp+motion | 2539 | 0 | 6 | wavelet-HHL_glszm_GrayLevelNonUniformity_motion | 0.785 |
| FEV₁ | 11_exp+expmax+motion | 2539 | 0 | 6 | wavelet-HHL_glszm_GrayLevelNonUniformity_motion | 0.795 |
| FEV₁ | 12_insp+inspmax+exp+expmax+motion | 4241 | 0 | 6 | wavelet-HHL_glszm_GrayLevelNonUniformity_motion | 0.665 |
| FVC | 01_insp | 851 | 3 | 10 | original_shape_MajorAxisLength_insp | 0.975 |
| FVC | 02_exp | 851 | 3 | 5 | original_shape_MajorAxisLength_exp | 0.960 |
| FVC | 03_insp+exp | 1702 | 2 | 12 | original_shape_Elongation_insp | 0.945 |
| FVC | 04_insp+inspmax | 1702 | 2 | 8 | original_shape_Elongation_insp | 0.955 |
| FVC | 05_inspmax+exp | 1702 | 1 | 8 | original_shape_MajorAxisLength_insp_max_diff | 0.970 |
| FVC | 06_exp+expmax | 1702 | 2 | 6 | wavelet-HHH_glszm_GrayLevelNonUniformity_exp_max_d | 0.870 |
| FVC | 07_insp+inspmax+exp+expmax | 3404 | 2 | 7 | original_shape_Elongation_insp | 0.915 |
| FVC | 08_insp+exp+motion | 2539 | 1 | 10 | wavelet-HHL_glszm_GrayLevelNonUniformity_motion | 0.820 |
| FVC | 09_insp+inspmax+motion | 2539 | 1 | 10 | wavelet-HHL_glszm_GrayLevelNonUniformity_motion | 0.855 |
| FVC | 10_inspmax+exp+motion | 2539 | 1 | 8 | wavelet-HHL_glszm_GrayLevelNonUniformity_motion | 0.875 |
| FVC | 11_exp+expmax+motion | 2539 | 1 | 5 | wavelet-HHL_glszm_GrayLevelNonUniformity_motion | 0.885 |
| FVC | 12_insp+inspmax+exp+expmax+motion | 4241 | 1 | 8 | wavelet-HHL_glszm_GrayLevelNonUniformity_motion | 0.825 |
| Disease (3-class) | 01_insp | 851 | 9 | 33 | wavelet-HHL_firstorder_Skewness_insp | 0.980 |
| Disease (3-class) | 02_exp | 851 | 8 | 24 | wavelet-HLL_glszm_ZoneEntropy_exp | 1.000 |
| Disease (3-class) | 03_insp+exp | 1702 | 4 | 27 | wavelet-HHL_firstorder_Skewness_insp | 0.925 |
| Disease (3-class) | 07_insp+inspmax+exp+expmax | 3404 | 5 | 31 | wavelet-HHL_firstorder_Median_insp_max_diff | 0.910 |
| Disease (3-class) | 08_insp+exp+motion | 2539 | 6 | 37 | wavelet-LHL_gldm_DependenceNonUniformityNormalized | 0.995 |
| Disease (3-class) | 12_insp+inspmax+exp+expmax+motion | 4241 | 5 | 44 | wavelet-LHL_gldm_DependenceNonUniformityNormalized | 0.985 |

***sTable 2. Robust radiomic features (selection frequency ≥ 0.80) for FEV₁ and FVC regression.***

| **Target** | **Combination** | **Feature** | **Frequency** | **Image type** | **Feature class** |
| --- | --- | --- | --- | --- | --- |
| FEV₁ | 01_insp | original_shape_MajorAxisLength_insp | 0.975 | insp | shape |
| FEV₁ | 01_insp | original_shape_Elongation_insp | 0.865 | insp | shape |
| FEV₁ | 02_exp | original_shape_MajorAxisLength_exp | 0.930 | exp | shape |
| FEV₁ | 02_exp | wavelet-HHH_glszm_GrayLevelNonUniformity_exp | 0.800 | exp | glszm |
| FEV₁ | 03_insp+exp | original_shape_MajorAxisLength_insp | 0.925 | insp | shape |
| FEV₁ | 03_insp+exp | original_shape_Elongation_insp | 0.865 | insp | shape |
| FEV₁ | 04_insp+inspmax | original_shape_MajorAxisLength_insp | 0.965 | insp | shape |
| FEV₁ | 04_insp+inspmax | original_shape_Elongation_insp | 0.845 | insp | shape |
| FEV₁ | 05_inspmax+exp | original_shape_MajorAxisLength_insp_max_diff | 0.935 | insp_max_diff | shape |
| FEV₁ | 06_exp+expmax | original_shape_MajorAxisLength_exp | 0.810 | exp | shape |
| FEV₁ | 07_insp+inspmax+exp+expmax | original_shape_MajorAxisLength_insp | 0.895 | insp | shape |
| FEV₁ | 07_insp+inspmax+exp+expmax | original_shape_Elongation_insp | 0.840 | insp | shape |
| FVC | 01_insp | original_shape_MajorAxisLength_insp | 0.975 | insp | shape |
| FVC | 01_insp | original_shape_Elongation_insp | 0.965 | insp | shape |
| FVC | 01_insp | wavelet-HHL_firstorder_Kurtosis_insp | 0.815 | insp | firstorder |
| FVC | 02_exp | original_shape_MajorAxisLength_exp | 0.960 | exp | shape |
| FVC | 02_exp | wavelet-HHH_glszm_GrayLevelNonUniformity_exp | 0.895 | exp | glszm |
| FVC | 02_exp | wavelet-HHL_glcm_Idmn_exp | 0.815 | exp | glcm |
| FVC | 03_insp+exp | original_shape_Elongation_insp | 0.945 | insp | shape |
| FVC | 03_insp+exp | original_shape_MajorAxisLength_insp | 0.920 | insp | shape |
| FVC | 04_insp+inspmax | original_shape_Elongation_insp | 0.955 | insp | shape |
| FVC | 04_insp+inspmax | original_shape_MajorAxisLength_insp | 0.945 | insp | shape |
| FVC | 05_inspmax+exp | original_shape_MajorAxisLength_insp_max_diff | 0.970 | insp_max_diff | shape |
| FVC | 06_exp+expmax | wavelet-HHH_glszm_GrayLevelNonUniformity_exp_max_diff | 0.870 | exp_max_diff | glszm |
| FVC | 06_exp+expmax | original_shape_MajorAxisLength_exp | 0.850 | exp | shape |
| FVC | 07_insp+inspmax+exp+expmax | original_shape_Elongation_insp | 0.915 | insp | shape |
| FVC | 07_insp+inspmax+exp+expmax | original_shape_MajorAxisLength_insp | 0.860 | insp | shape |
| FVC | 08_insp+exp+motion | wavelet-HHL_glszm_GrayLevelNonUniformity_motion | 0.820 | motion | glszm |
| FVC | 09_insp+inspmax+motion | wavelet-HHL_glszm_GrayLevelNonUniformity_motion | 0.855 | motion | glszm |
| FVC | 10_inspmax+exp+motion | wavelet-HHL_glszm_GrayLevelNonUniformity_motion | 0.875 | motion | glszm |
| FVC | 11_exp+expmax+motion | wavelet-HHL_glszm_GrayLevelNonUniformity_motion | 0.885 | motion | glszm |
| FVC | 12_insp+inspmax+exp+expmax+motion | wavelet-HHL_glszm_GrayLevelNonUniformity_motion | 0.825 | motion | glszm |

***sTable 3. Robust radiomic features (selection frequency ≥ 0.80) for 3-class disease classification.***

| **Combination** | **Feature** | **Frequency** |
| --- | --- | --- |
| 01_insp | wavelet-HHL_firstorder_Skewness_insp | 0.980 |
| 01_insp | wavelet-HLL_glszm_ZoneEntropy_insp | 0.935 |
| 01_insp | wavelet-LHL_firstorder_Median_insp | 0.900 |
| 01_insp | wavelet-LHL_glcm_Correlation_insp | 0.845 |
| 01_insp | wavelet-LHL_ngtdm_Contrast_insp | 0.830 |
| 01_insp | wavelet-HHH_glszm_GrayLevelNonUniformity_insp | 0.815 |
| 01_insp | wavelet-LHH_glszm_GrayLevelNonUniformityNormalized_insp | 0.810 |
| 01_insp | wavelet-HLL_firstorder_Median_insp | 0.810 |
| 01_insp | wavelet-LHL_glcm_ClusterShade_insp | 0.800 |
| 02_exp | wavelet-HLL_glszm_ZoneEntropy_exp | 1.000 |
| 02_exp | wavelet-HHL_firstorder_Kurtosis_exp | 0.965 |
| 02_exp | original_firstorder_90Percentile_exp | 0.960 |
| 02_exp | original_ngtdm_Busyness_exp | 0.900 |
| 02_exp | original_firstorder_10Percentile_exp | 0.885 |
| 02_exp | original_shape_Elongation_exp | 0.840 |
| 02_exp | wavelet-LHL_glcm_Correlation_exp | 0.835 |
| 02_exp | original_glcm_Contrast_exp | 0.835 |
| 03_insp+exp | wavelet-HHL_firstorder_Skewness_insp | 0.925 |
| 03_insp+exp | original_firstorder_90Percentile_exp | 0.880 |
| 03_insp+exp | wavelet-HHL_firstorder_Kurtosis_exp | 0.835 |
| 03_insp+exp | wavelet-HLL_glszm_ZoneEntropy_insp | 0.805 |
| 07_insp+inspmax+exp+expmax | wavelet-HHL_firstorder_Median_insp_max_diff | 0.910 |
| 07_insp+inspmax+exp+expmax | wavelet-HLL_glszm_ZoneEntropy_insp | 0.860 |
| 07_insp+inspmax+exp+expmax | wavelet-HHL_firstorder_Skewness_insp | 0.850 |
| 07_insp+inspmax+exp+expmax | wavelet-LHL_firstorder_Median_insp_max_diff | 0.805 |
| 07_insp+inspmax+exp+expmax | wavelet-LHL_firstorder_Skewness_insp_max_diff | 0.800 |
| 08_insp+exp+motion | wavelet-LHL_gldm_DependenceNonUniformityNormalized_motion | 0.995 |
| 08_insp+exp+motion | wavelet-HHL_firstorder_Skewness_insp | 0.950 |
| 08_insp+exp+motion | original_firstorder_90Percentile_exp | 0.900 |
| 08_insp+exp+motion | wavelet-HLL_glszm_ZoneEntropy_insp | 0.870 |
| 08_insp+exp+motion | original_glcm_Contrast_motion | 0.815 |
| 08_insp+exp+motion | wavelet-HHL_firstorder_Kurtosis_exp | 0.800 |
| 12_insp+inspmax+exp+expmax+motion | wavelet-LHL_gldm_DependenceNonUniformityNormalized_motion | 0.985 |
| 12_insp+inspmax+exp+expmax+motion | wavelet-HLL_glszm_ZoneEntropy_insp | 0.880 |
| 12_insp+inspmax+exp+expmax+motion | wavelet-HHL_firstorder_Skewness_insp | 0.870 |
| 12_insp+inspmax+exp+expmax+motion | wavelet-HHL_firstorder_Median_insp_max_diff | 0.850 |
| 12_insp+inspmax+exp+expmax+motion | wavelet-LHL_firstorder_Median_exp_max_diff | 0.815 |

***sTable 4. Multi-seed × multi-algorithm prediction performance for FEV₁ and FVC over 50 random 70 : 30 splits.***

| **Target** | **Phase** | **Combination** | **Algorithm** | **n features** | **R mean** | **R 2.5 %** | **R 97.5 %** | **MAE mean** | **MAE 2.5 %** | **MAE 97.5 %** |
| --- | --- | --- | --- | --- | --- | --- | --- | --- | --- | --- |
| FEV₁ | 0_full | 01_insp | LASSO | 9.620 | 0.520 | 0.292 | 0.709 | 0.431 | 0.344 | 0.546 |
| FEV₁ | 0_full | 01_insp | Linear | 9.620 | 0.515 | 0.278 | 0.692 | 0.435 | 0.346 | 0.572 |
| FEV₁ | 0_full | 01_insp | RandomForest | 9.620 | 0.524 | 0.292 | 0.663 | 0.425 | 0.336 | 0.521 |
| FEV₁ | 0_full | 01_insp | Ridge | 9.620 | 0.511 | 0.281 | 0.675 | 0.432 | 0.348 | 0.534 |
| FEV₁ | 0_full | 02_exp | LASSO | 7.660 | 0.315 | 0.069 | 0.527 | 0.494 | 0.381 | 0.685 |
| FEV₁ | 0_full | 02_exp | Linear | 7.660 | 0.309 | 0.071 | 0.526 | 0.504 | 0.385 | 0.706 |
| FEV₁ | 0_full | 02_exp | RandomForest | 7.660 | 0.321 | 0.106 | 0.549 | 0.470 | 0.375 | 0.542 |
| FEV₁ | 0_full | 02_exp | Ridge | 7.660 | 0.314 | 0.038 | 0.528 | 0.486 | 0.384 | 0.645 |
| FEV₁ | 0_full | 03_insp+exp | LASSO | 12.580 | 0.478 | 0.241 | 0.648 | 0.449 | 0.363 | 0.576 |
| FEV₁ | 0_full | 03_insp+exp | Linear | 12.580 | 0.463 | 0.243 | 0.631 | 0.461 | 0.369 | 0.606 |
| FEV₁ | 0_full | 03_insp+exp | RandomForest | 12.580 | 0.501 | 0.272 | 0.658 | 0.428 | 0.336 | 0.507 |
| FEV₁ | 0_full | 03_insp+exp | Ridge | 12.580 | 0.466 | 0.240 | 0.649 | 0.455 | 0.376 | 0.557 |
| FEV₁ | 0_full | 07_insp+inspmax+exp+expmax | LASSO | 16.680 | 0.454 | 0.047 | 0.671 | 0.466 | 0.343 | 0.639 |
| FEV₁ | 0_full | 07_insp+inspmax+exp+expmax | Linear | 16.680 | 0.443 | 0.027 | 0.656 | 0.478 | 0.375 | 0.664 |
| FEV₁ | 0_full | 07_insp+inspmax+exp+expmax | RandomForest | 16.680 | 0.470 | 0.201 | 0.669 | 0.437 | 0.344 | 0.522 |
| FEV₁ | 0_full | 07_insp+inspmax+exp+expmax | Ridge | 16.680 | 0.433 | 0.150 | 0.658 | 0.468 | 0.358 | 0.603 |
| FEV₁ | 0_full | 08_insp+exp+motion | LASSO | 15.840 | 0.448 | 0.115 | 0.669 | 0.475 | 0.382 | 0.743 |
| FEV₁ | 0_full | 08_insp+exp+motion | Linear | 15.840 | 0.432 | 0.110 | 0.665 | 0.488 | 0.391 | 0.775 |
| FEV₁ | 0_full | 08_insp+exp+motion | RandomForest | 15.840 | 0.537 | 0.303 | 0.722 | 0.419 | 0.330 | 0.491 |
| FEV₁ | 0_full | 08_insp+exp+motion | Ridge | 15.840 | 0.479 | 0.152 | 0.696 | 0.453 | 0.344 | 0.617 |
| FEV₁ | 0_full | 12_insp+inspmax+exp+expmax+motion | LASSO | 20.280 | 0.425 | 0.044 | 0.666 | 0.489 | 0.369 | 0.735 |
| FEV₁ | 0_full | 12_insp+inspmax+exp+expmax+motion | Linear | 20.280 | 0.399 | 0.028 | 0.650 | 0.507 | 0.386 | 0.782 |
| FEV₁ | 0_full | 12_insp+inspmax+exp+expmax+motion | RandomForest | 20.280 | 0.527 | 0.301 | 0.680 | 0.424 | 0.360 | 0.506 |
| FEV₁ | 0_full | 12_insp+inspmax+exp+expmax+motion | Ridge | 20.280 | 0.453 | 0.052 | 0.654 | 0.474 | 0.353 | 0.720 |
| FEV₁ | A_robust | 01_insp | LASSO | 2.000 | 0.609 | 0.383 | 0.754 | 0.381 | 0.306 | 0.446 |
| FEV₁ | A_robust | 01_insp | Linear | 2.000 | 0.609 | 0.383 | 0.756 | 0.381 | 0.306 | 0.446 |
| FEV₁ | A_robust | 01_insp | RandomForest | 2.000 | 0.543 | 0.309 | 0.742 | 0.411 | 0.341 | 0.516 |
| FEV₁ | A_robust | 01_insp | Ridge | 2.000 | 0.609 | 0.383 | 0.755 | 0.386 | 0.309 | 0.466 |
| FEV₁ | A_robust | 02_exp | LASSO | 2.000 | 0.388 | 0.088 | 0.581 | 0.466 | 0.365 | 0.541 |
| FEV₁ | A_robust | 02_exp | Linear | 2.000 | 0.391 | 0.091 | 0.586 | 0.465 | 0.363 | 0.545 |
| FEV₁ | A_robust | 02_exp | RandomForest | 2.000 | 0.293 | 0.049 | 0.555 | 0.491 | 0.403 | 0.575 |
| FEV₁ | A_robust | 02_exp | Ridge | 2.000 | 0.392 | 0.092 | 0.592 | 0.465 | 0.365 | 0.533 |
| FEV₁ | A_robust | 03_insp+exp | LASSO | 2.000 | 0.609 | 0.383 | 0.754 | 0.381 | 0.306 | 0.446 |
| FEV₁ | A_robust | 03_insp+exp | Linear | 2.000 | 0.609 | 0.383 | 0.756 | 0.381 | 0.306 | 0.446 |
| FEV₁ | A_robust | 03_insp+exp | RandomForest | 2.000 | 0.543 | 0.309 | 0.742 | 0.411 | 0.341 | 0.516 |
| FEV₁ | A_robust | 03_insp+exp | Ridge | 2.000 | 0.609 | 0.383 | 0.755 | 0.386 | 0.309 | 0.466 |
| FEV₁ | A_robust | 07_insp+inspmax+exp+expmax | LASSO | 2.000 | 0.609 | 0.383 | 0.754 | 0.381 | 0.306 | 0.446 |
| FEV₁ | A_robust | 07_insp+inspmax+exp+expmax | Linear | 2.000 | 0.609 | 0.383 | 0.756 | 0.381 | 0.306 | 0.446 |
| FEV₁ | A_robust | 07_insp+inspmax+exp+expmax | RandomForest | 2.000 | 0.543 | 0.309 | 0.742 | 0.411 | 0.341 | 0.516 |
| FEV₁ | A_robust | 07_insp+inspmax+exp+expmax | Ridge | 2.000 | 0.609 | 0.383 | 0.755 | 0.386 | 0.309 | 0.466 |
| FEV₁ | A_robust | 08_insp+exp+motion | LASSO | 0.00e+00 |  |  |  |  |  |  |
| FEV₁ | A_robust | 08_insp+exp+motion | Linear | 0.00e+00 |  |  |  |  |  |  |
| FEV₁ | A_robust | 08_insp+exp+motion | RandomForest | 0.00e+00 |  |  |  |  |  |  |
| FEV₁ | A_robust | 08_insp+exp+motion | Ridge | 0.00e+00 |  |  |  |  |  |  |
| FEV₁ | A_robust | 12_insp+inspmax+exp+expmax+motion | LASSO | 0.00e+00 |  |  |  |  |  |  |
| FEV₁ | A_robust | 12_insp+inspmax+exp+expmax+motion | Linear | 0.00e+00 |  |  |  |  |  |  |
| FEV₁ | A_robust | 12_insp+inspmax+exp+expmax+motion | RandomForest | 0.00e+00 |  |  |  |  |  |  |
| FEV₁ | A_robust | 12_insp+inspmax+exp+expmax+motion | Ridge | 0.00e+00 |  |  |  |  |  |  |
| FEV₁ | B_Universal-2 | universal | LASSO | 2.000 | 0.609 | 0.383 | 0.754 | 0.381 | 0.306 | 0.446 |
| FEV₁ | B_Universal-2 | universal | Linear | 2.000 | 0.609 | 0.383 | 0.756 | 0.381 | 0.306 | 0.446 |
| FEV₁ | B_Universal-2 | universal | RandomForest | 2.000 | 0.543 | 0.309 | 0.742 | 0.411 | 0.341 | 0.516 |
| FEV₁ | B_Universal-2 | universal | Ridge | 2.000 | 0.609 | 0.383 | 0.755 | 0.386 | 0.309 | 0.466 |
| FEV₁ | B_Universal-3 | universal | LASSO | 3.000 | 0.603 | 0.386 | 0.754 | 0.385 | 0.307 | 0.468 |
| FEV₁ | B_Universal-3 | universal | Linear | 3.000 | 0.607 | 0.399 | 0.760 | 0.381 | 0.297 | 0.472 |
| FEV₁ | B_Universal-3 | universal | RandomForest | 3.000 | 0.521 | 0.234 | 0.715 | 0.417 | 0.349 | 0.516 |
| FEV₁ | B_Universal-3 | universal | Ridge | 3.000 | 0.605 | 0.382 | 0.753 | 0.389 | 0.326 | 0.476 |
| FVC | 0_full | 01_insp | LASSO | 12.900 | 0.720 | 0.545 | 0.837 | 0.455 | 0.342 | 0.591 |
| FVC | 0_full | 01_insp | Linear | 12.900 | 0.716 | 0.551 | 0.842 | 0.461 | 0.344 | 0.614 |
| FVC | 0_full | 01_insp | RandomForest | 12.900 | 0.701 | 0.554 | 0.832 | 0.487 | 0.380 | 0.603 |
| FVC | 0_full | 01_insp | Ridge | 12.900 | 0.716 | 0.530 | 0.840 | 0.459 | 0.349 | 0.578 |
| FVC | 0_full | 02_exp | LASSO | 8.740 | 0.453 | 0.178 | 0.660 | 0.616 | 0.483 | 0.870 |
| FVC | 0_full | 02_exp | Linear | 8.740 | 0.443 | 0.171 | 0.672 | 0.623 | 0.485 | 0.876 |
| FVC | 0_full | 02_exp | RandomForest | 8.740 | 0.511 | 0.316 | 0.655 | 0.583 | 0.491 | 0.669 |
| FVC | 0_full | 02_exp | Ridge | 8.740 | 0.468 | 0.179 | 0.683 | 0.611 | 0.505 | 0.854 |
| FVC | 0_full | 03_insp+exp | LASSO | 16.680 | 0.680 | 0.472 | 0.815 | 0.488 | 0.386 | 0.672 |
| FVC | 0_full | 03_insp+exp | Linear | 16.680 | 0.671 | 0.464 | 0.813 | 0.504 | 0.397 | 0.685 |
| FVC | 0_full | 03_insp+exp | RandomForest | 16.680 | 0.676 | 0.524 | 0.823 | 0.503 | 0.404 | 0.600 |
| FVC | 0_full | 03_insp+exp | Ridge | 16.680 | 0.671 | 0.462 | 0.810 | 0.495 | 0.401 | 0.658 |
| FVC | 0_full | 07_insp+inspmax+exp+expmax | LASSO | 20.300 | 0.647 | 0.399 | 0.812 | 0.517 | 0.410 | 0.754 |
| FVC | 0_full | 07_insp+inspmax+exp+expmax | Linear | 20.300 | 0.637 | 0.384 | 0.797 | 0.537 | 0.409 | 0.776 |
| FVC | 0_full | 07_insp+inspmax+exp+expmax | RandomForest | 20.300 | 0.660 | 0.440 | 0.797 | 0.514 | 0.400 | 0.638 |
| FVC | 0_full | 07_insp+inspmax+exp+expmax | Ridge | 20.300 | 0.631 | 0.344 | 0.781 | 0.518 | 0.409 | 0.687 |
| FVC | 0_full | 08_insp+exp+motion | LASSO | 19.160 | 0.663 | 0.013 | 0.855 | 0.521 | 0.365 | 1.284 |
| FVC | 0_full | 08_insp+exp+motion | Linear | 19.160 | 0.651 | 0.012 | 0.849 | 0.536 | 0.369 | 1.304 |
| FVC | 0_full | 08_insp+exp+motion | RandomForest | 19.160 | 0.740 | 0.574 | 0.851 | 0.441 | 0.328 | 0.519 |
| FVC | 0_full | 08_insp+exp+motion | Ridge | 19.160 | 0.660 | 0.042 | 0.858 | 0.502 | 0.357 | 0.950 |
| FVC | 0_full | 12_insp+inspmax+exp+expmax+motion | LASSO | 20.580 | 0.651 | -0.002 | 0.845 | 0.540 | 0.380 | 1.334 |
| FVC | 0_full | 12_insp+inspmax+exp+expmax+motion | Linear | 20.580 | 0.637 | -0.005 | 0.841 | 0.562 | 0.387 | 1.379 |
| FVC | 0_full | 12_insp+inspmax+exp+expmax+motion | RandomForest | 20.580 | 0.737 | 0.542 | 0.858 | 0.445 | 0.351 | 0.549 |
| FVC | 0_full | 12_insp+inspmax+exp+expmax+motion | Ridge | 20.580 | 0.656 | 0.027 | 0.846 | 0.516 | 0.365 | 0.937 |
| FVC | A_robust | 01_insp | LASSO | 3.000 | 0.750 | 0.585 | 0.874 | 0.425 | 0.337 | 0.511 |
| FVC | A_robust | 01_insp | Linear | 3.000 | 0.751 | 0.585 | 0.874 | 0.425 | 0.337 | 0.511 |
| FVC | A_robust | 01_insp | RandomForest | 3.000 | 0.707 | 0.573 | 0.836 | 0.470 | 0.367 | 0.561 |
| FVC | A_robust | 01_insp | Ridge | 3.000 | 0.750 | 0.575 | 0.873 | 0.428 | 0.346 | 0.530 |
| FVC | A_robust | 02_exp | LASSO | 3.000 | 0.497 | 0.277 | 0.668 | 0.595 | 0.479 | 0.707 |
| FVC | A_robust | 02_exp | Linear | 3.000 | 0.507 | 0.295 | 0.681 | 0.589 | 0.468 | 0.687 |
| FVC | A_robust | 02_exp | RandomForest | 3.000 | 0.475 | 0.241 | 0.668 | 0.599 | 0.488 | 0.701 |
| FVC | A_robust | 02_exp | Ridge | 3.000 | 0.514 | 0.305 | 0.686 | 0.592 | 0.497 | 0.716 |
| FVC | A_robust | 03_insp+exp | LASSO | 2.000 | 0.741 | 0.610 | 0.855 | 0.442 | 0.341 | 0.529 |
| FVC | A_robust | 03_insp+exp | Linear | 2.000 | 0.741 | 0.610 | 0.855 | 0.442 | 0.342 | 0.526 |
| FVC | A_robust | 03_insp+exp | RandomForest | 2.000 | 0.715 | 0.575 | 0.828 | 0.466 | 0.371 | 0.556 |
| FVC | A_robust | 03_insp+exp | Ridge | 2.000 | 0.741 | 0.610 | 0.855 | 0.444 | 0.329 | 0.546 |
| FVC | A_robust | 07_insp+inspmax+exp+expmax | LASSO | 2.000 | 0.741 | 0.610 | 0.855 | 0.442 | 0.341 | 0.529 |
| FVC | A_robust | 07_insp+inspmax+exp+expmax | Linear | 2.000 | 0.741 | 0.610 | 0.855 | 0.442 | 0.342 | 0.526 |
| FVC | A_robust | 07_insp+inspmax+exp+expmax | RandomForest | 2.000 | 0.715 | 0.575 | 0.828 | 0.466 | 0.371 | 0.556 |
| FVC | A_robust | 07_insp+inspmax+exp+expmax | Ridge | 2.000 | 0.741 | 0.610 | 0.855 | 0.444 | 0.329 | 0.546 |
| FVC | A_robust | 08_insp+exp+motion | LASSO | 1.000 | 0.594 | 0.367 | 0.799 | 0.542 | 0.442 | 0.675 |
| FVC | A_robust | 08_insp+exp+motion | Linear | 1.000 | 0.594 | 0.367 | 0.799 | 0.539 | 0.433 | 0.675 |
| FVC | A_robust | 08_insp+exp+motion | RandomForest | 1.000 | 0.577 | 0.405 | 0.714 | 0.551 | 0.427 | 0.664 |
| FVC | A_robust | 08_insp+exp+motion | Ridge | 1.000 | 0.594 | 0.367 | 0.799 | 0.558 | 0.453 | 0.676 |
| FVC | A_robust | 12_insp+inspmax+exp+expmax+motion | LASSO | 1.000 | 0.594 | 0.367 | 0.799 | 0.542 | 0.442 | 0.675 |
| FVC | A_robust | 12_insp+inspmax+exp+expmax+motion | Linear | 1.000 | 0.594 | 0.367 | 0.799 | 0.539 | 0.433 | 0.675 |
| FVC | A_robust | 12_insp+inspmax+exp+expmax+motion | RandomForest | 1.000 | 0.577 | 0.405 | 0.714 | 0.551 | 0.427 | 0.664 |
| FVC | A_robust | 12_insp+inspmax+exp+expmax+motion | Ridge | 1.000 | 0.594 | 0.367 | 0.799 | 0.558 | 0.453 | 0.676 |
| FVC | B_Universal-2 | universal | LASSO | 2.000 | 0.741 | 0.610 | 0.855 | 0.442 | 0.341 | 0.529 |
| FVC | B_Universal-2 | universal | Linear | 2.000 | 0.741 | 0.610 | 0.855 | 0.442 | 0.342 | 0.526 |
| FVC | B_Universal-2 | universal | RandomForest | 2.000 | 0.715 | 0.574 | 0.828 | 0.466 | 0.370 | 0.555 |
| FVC | B_Universal-2 | universal | Ridge | 2.000 | 0.741 | 0.610 | 0.855 | 0.444 | 0.329 | 0.546 |
| FVC | B_Universal-3 | universal | LASSO | 3.000 | 0.743 | 0.626 | 0.854 | 0.444 | 0.360 | 0.547 |
| FVC | B_Universal-3 | universal | Linear | 3.000 | 0.742 | 0.626 | 0.860 | 0.445 | 0.362 | 0.548 |
| FVC | B_Universal-3 | universal | RandomForest | 3.000 | 0.692 | 0.529 | 0.811 | 0.481 | 0.393 | 0.568 |
| FVC | B_Universal-3 | universal | Ridge | 3.000 | 0.739 | 0.623 | 0.848 | 0.447 | 0.331 | 0.543 |

***sTable 5. Multi-seed × multi-algorithm prediction performance for 3-class disease classification (Asthma vs COPD vs IP).***

| **Phase** | **Combination** | **Algorithm** | **n features** | **Accuracy** | **Macro F1** | **Macro AUC** | **AUC 2.5 %** | **AUC 97.5 %** |
| --- | --- | --- | --- | --- | --- | --- | --- | --- |
| 0_full | 01_insp | LASSO | 21.260 | 0.561 | 0.543 | 0.725 | 0.582 | 0.810 |
| 0_full | 01_insp | LogReg | 21.260 | 0.525 | 0.507 | 0.683 | 0.537 | 0.799 |
| 0_full | 01_insp | RandomForest | 21.260 | 0.634 | 0.591 | 0.779 | 0.693 | 0.875 |
| 0_full | 01_insp | Ridge | 21.260 | 0.568 | 0.554 | 0.755 | 0.631 | 0.846 |
| 0_full | 02_exp | LASSO | 16.720 | 0.503 | 0.478 | 0.697 | 0.599 | 0.829 |
| 0_full | 02_exp | LogReg | 16.720 | 0.508 | 0.477 | 0.673 | 0.558 | 0.835 |
| 0_full | 02_exp | RandomForest | 16.720 | 0.592 | 0.543 | 0.741 | 0.629 | 0.828 |
| 0_full | 02_exp | Ridge | 16.720 | 0.510 | 0.490 | 0.713 | 0.610 | 0.825 |
| 0_full | 03_insp+exp | LASSO | 33.480 | 0.517 | 0.493 | 0.691 | 0.572 | 0.789 |
| 0_full | 03_insp+exp | LogReg | 33.480 | 0.487 | 0.460 | 0.653 | 0.535 | 0.758 |
| 0_full | 03_insp+exp | RandomForest | 33.480 | 0.616 | 0.561 | 0.767 | 0.669 | 0.860 |
| 0_full | 03_insp+exp | Ridge | 33.480 | 0.539 | 0.523 | 0.738 | 0.614 | 0.849 |
| 0_full | 07_insp+inspmax+exp+expmax | LASSO | 44.940 | 0.544 | 0.513 | 0.716 | 0.615 | 0.826 |
| 0_full | 07_insp+inspmax+exp+expmax | LogReg | 44.940 | 0.537 | 0.500 | 0.696 | 0.581 | 0.797 |
| 0_full | 07_insp+inspmax+exp+expmax | RandomForest | 44.940 | 0.616 | 0.559 | 0.771 | 0.683 | 0.848 |
| 0_full | 07_insp+inspmax+exp+expmax | Ridge | 44.940 | 0.555 | 0.533 | 0.754 | 0.661 | 0.863 |
| 0_full | 08_insp+exp+motion | LASSO | 53.860 | 0.582 | 0.548 | 0.745 | 0.623 | 0.850 |
| 0_full | 08_insp+exp+motion | LogReg | 53.860 | 0.582 | 0.548 | 0.736 | 0.626 | 0.832 |
| 0_full | 08_insp+exp+motion | RandomForest | 53.860 | 0.634 | 0.564 | 0.784 | 0.685 | 0.877 |
| 0_full | 08_insp+exp+motion | Ridge | 53.860 | 0.592 | 0.558 | 0.766 | 0.641 | 0.866 |
| 0_full | 12_insp+inspmax+exp+expmax+motion | LASSO | 79.380 | 0.584 | 0.547 | 0.743 | 0.611 | 0.847 |
| 0_full | 12_insp+inspmax+exp+expmax+motion | LogReg | 79.380 | 0.565 | 0.526 | 0.730 | 0.619 | 0.855 |
| 0_full | 12_insp+inspmax+exp+expmax+motion | RandomForest | 79.380 | 0.627 | 0.555 | 0.784 | 0.709 | 0.860 |
| 0_full | 12_insp+inspmax+exp+expmax+motion | Ridge | 79.380 | 0.592 | 0.554 | 0.772 | 0.632 | 0.871 |
| A_robust | 01_insp | LASSO | 9.000 | 0.572 | 0.554 | 0.775 | 0.673 | 0.899 |
| A_robust | 01_insp | LogReg | 9.000 | 0.574 | 0.555 | 0.765 | 0.641 | 0.886 |
| A_robust | 01_insp | RandomForest | 9.000 | 0.637 | 0.598 | 0.789 | 0.702 | 0.896 |
| A_robust | 01_insp | Ridge | 9.000 | 0.630 | 0.613 | 0.809 | 0.737 | 0.907 |
| A_robust | 02_exp | LASSO | 8.000 | 0.578 | 0.557 | 0.776 | 0.690 | 0.853 |
| A_robust | 02_exp | LogReg | 8.000 | 0.566 | 0.546 | 0.770 | 0.682 | 0.852 |
| A_robust | 02_exp | RandomForest | 8.000 | 0.619 | 0.570 | 0.769 | 0.682 | 0.834 |
| A_robust | 02_exp | Ridge | 8.000 | 0.588 | 0.573 | 0.790 | 0.690 | 0.872 |
| A_robust | 03_insp+exp | LASSO | 4.000 | 0.625 | 0.608 | 0.787 | 0.706 | 0.869 |
| A_robust | 03_insp+exp | LogReg | 4.000 | 0.606 | 0.590 | 0.786 | 0.706 | 0.878 |
| A_robust | 03_insp+exp | RandomForest | 4.000 | 0.597 | 0.550 | 0.741 | 0.655 | 0.827 |
| A_robust | 03_insp+exp | Ridge | 4.000 | 0.630 | 0.615 | 0.788 | 0.723 | 0.856 |
| A_robust | 07_insp+inspmax+exp+expmax | LASSO | 5.000 | 0.588 | 0.573 | 0.778 | 0.685 | 0.885 |
| A_robust | 07_insp+inspmax+exp+expmax | LogReg | 5.000 | 0.577 | 0.565 | 0.773 | 0.675 | 0.875 |
| A_robust | 07_insp+inspmax+exp+expmax | RandomForest | 5.000 | 0.579 | 0.523 | 0.735 | 0.641 | 0.855 |
| A_robust | 07_insp+inspmax+exp+expmax | Ridge | 5.000 | 0.614 | 0.594 | 0.783 | 0.693 | 0.862 |
| A_robust | 08_insp+exp+motion | LASSO | 6.000 | 0.599 | 0.583 | 0.782 | 0.699 | 0.870 |
| A_robust | 08_insp+exp+motion | LogReg | 6.000 | 0.585 | 0.568 | 0.776 | 0.689 | 0.868 |
| A_robust | 08_insp+exp+motion | RandomForest | 6.000 | 0.578 | 0.515 | 0.746 | 0.678 | 0.811 |
| A_robust | 08_insp+exp+motion | Ridge | 6.000 | 0.617 | 0.597 | 0.790 | 0.704 | 0.869 |
| A_robust | 12_insp+inspmax+exp+expmax+motion | LASSO | 5.000 | 0.572 | 0.554 | 0.785 | 0.683 | 0.867 |
| A_robust | 12_insp+inspmax+exp+expmax+motion | LogReg | 5.000 | 0.570 | 0.554 | 0.788 | 0.688 | 0.877 |
| A_robust | 12_insp+inspmax+exp+expmax+motion | RandomForest | 5.000 | 0.581 | 0.533 | 0.750 | 0.654 | 0.834 |
| A_robust | 12_insp+inspmax+exp+expmax+motion | Ridge | 5.000 | 0.603 | 0.575 | 0.790 | 0.715 | 0.856 |
| B_Universal-2 | universal | LASSO | 2.000 | 0.422 | 0.391 | 0.611 | 0.500 | 0.731 |
| B_Universal-2 | universal | LogReg | 2.000 | 0.446 | 0.446 | 0.642 | 0.544 | 0.770 |
| B_Universal-2 | universal | RandomForest | 2.000 | 0.486 | 0.431 | 0.612 | 0.542 | 0.703 |
| B_Universal-2 | universal | Ridge | 2.000 | 0.438 | 0.437 | 0.629 | 0.532 | 0.752 |
| B_Universal-3 | universal | LASSO | 3.000 | 0.479 | 0.474 | 0.647 | 0.568 | 0.746 |
| B_Universal-3 | universal | LogReg | 3.000 | 0.477 | 0.473 | 0.650 | 0.567 | 0.750 |
| B_Universal-3 | universal | RandomForest | 3.000 | 0.513 | 0.457 | 0.651 | 0.582 | 0.754 |
| B_Universal-3 | universal | Ridge | 3.000 | 0.469 | 0.465 | 0.646 | 0.560 | 0.759 |

***sTable 6. Three-class disease classification performance of spirometry versus DCR radiomics over 50 stratified 70 : 30 splits (best algorithm per model; mean and 95 % CI). ΔAUC and the paired Wilcoxon signed-rank p value are relative to the DCR radiomic model. The full spirometry panel is included as a reference; it additionally encodes age/sex/height through the percent-predicted values, information not available to the radiograph.***

| **Model** | **Algorithm** | **Macro AUC (95 % CI)** | **Accuracy** | **Macro F1** | **ΔAUC vs DCR** | **Paired p vs DCR** |
| --- | --- | --- | --- | --- | --- | --- |
| DCR radiomics (9 robust EI features) | Ridge | 0.81 (0.74–0.91) | 0.63 | 0.61 | reference | — |
| FEV1 only | LogReg | 0.54 (0.41–0.65) | 0.33 | 0.29 | −0.27 | <0.001 |
| FVC only | RandomForest | 0.61 (0.52–0.71) | 0.49 | 0.45 | −0.20 | <0.001 |
| FEV1 + FVC | LogReg | 0.74 (0.66–0.83) | 0.53 | 0.53 | −0.07 | <0.001 |
| Full spirometry panel (reference) | LASSO | 0.83 (0.72–0.93) | 0.69 | 0.68 | +0.02 | 0.103 |
