# Supplementary material for: Estimation of pulmonary function from time‐resolved dynamic chest radiography using machine learning in patients with respiratory disease
Source: J Appl Clin Med Phys. 2026 Jul 27;27(8):e70717. doi: 10.1002/acm2.70717 (PMC13404249; doi:10.1002/acm2.70717)

DCR-radiomic prediction of FEV1, FVC, and disease class —
bootstrap stability (top row) and multi-algorithm performance (bottom row, mean ± 95 % CI over 50 random 70:30 splits)
P0 = full feature-selection pipeline · PA = robust-only features (bootstrap frequency ≥ 80 %) · U2 / U3 = Universal-2 / Universal-3 minimal-shape model

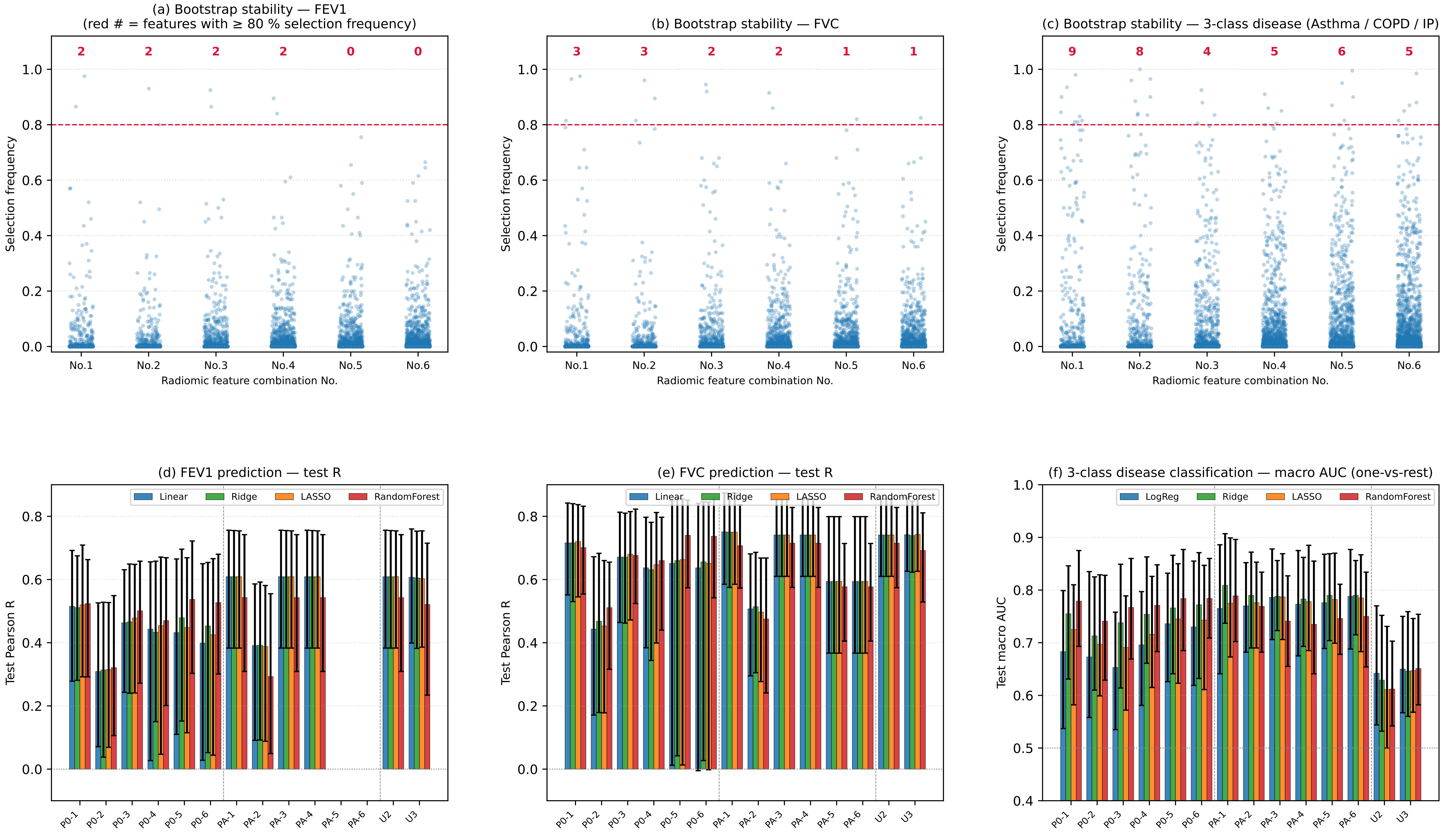

Supplement: Supplementary file 1 — Supporting Information [file ACM2-27-e70717-s001.zip › 2026-09352-sup-0005--S.pdf]
