# Supplementary figures and images for: Estimation of pulmonary function from time‐resolved dynamic chest radiography using machine learning in patients with respiratory disease
Source: J Appl Clin Med Phys. 2026 Jul 27;27(8):e70717. doi: 10.1002/acm2.70717 (PMC13404249; doi:10.1002/acm2.70717)

# Three-class disease classification (asthma vs COPD vs IP): DCR radiomics versus spirometry

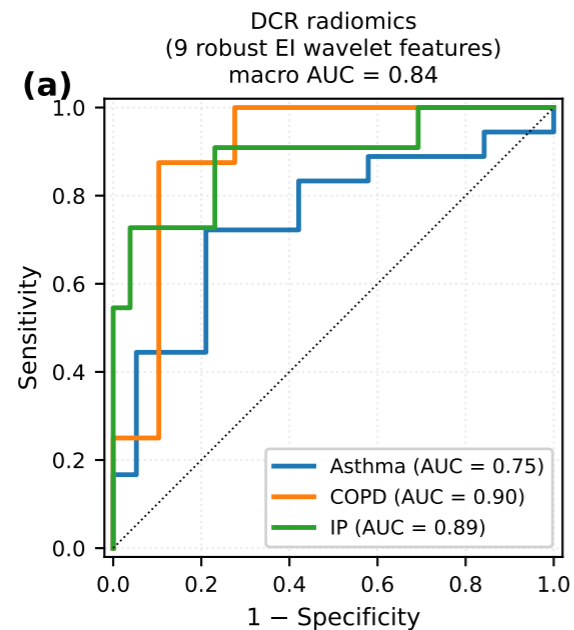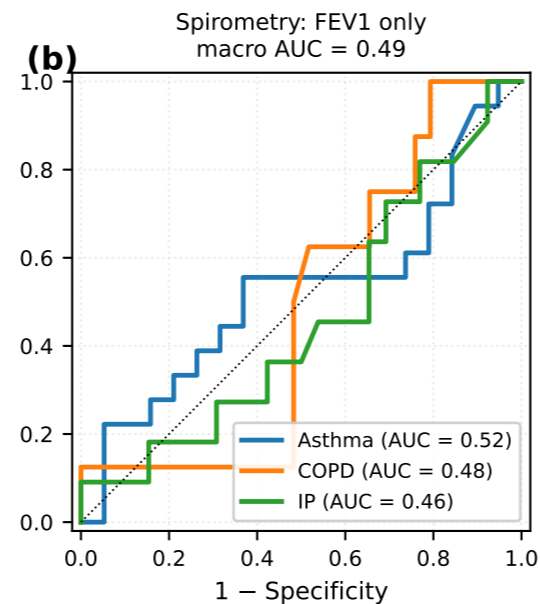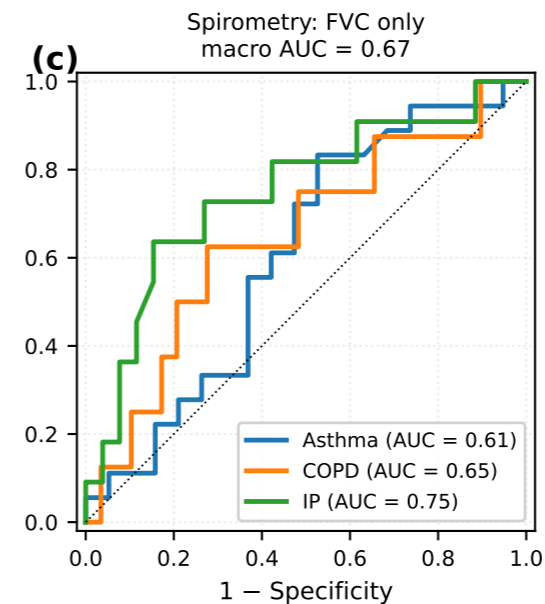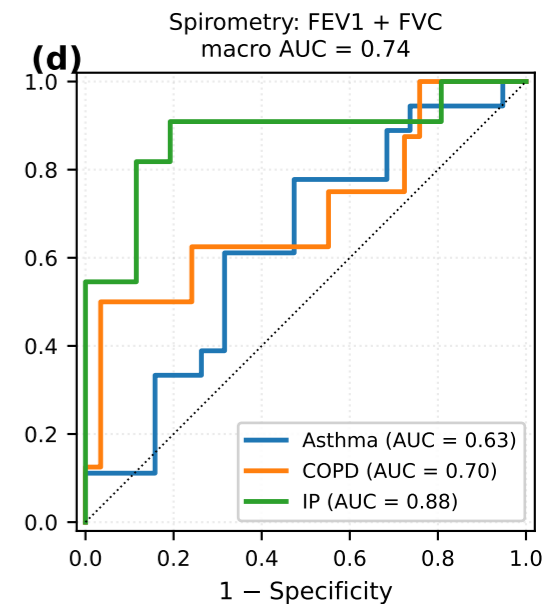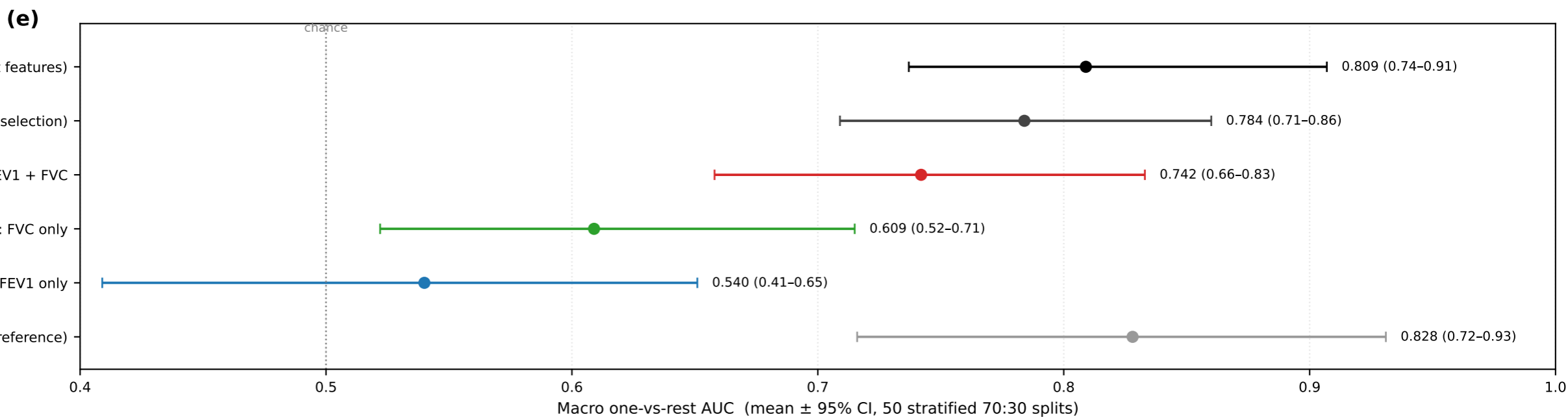

Supplement: Supplementary file 1 — Supporting Information [file ACM2-27-e70717-s001.zip › 2026-09352-sup-0006--S.pdf]

**sFigure 1**

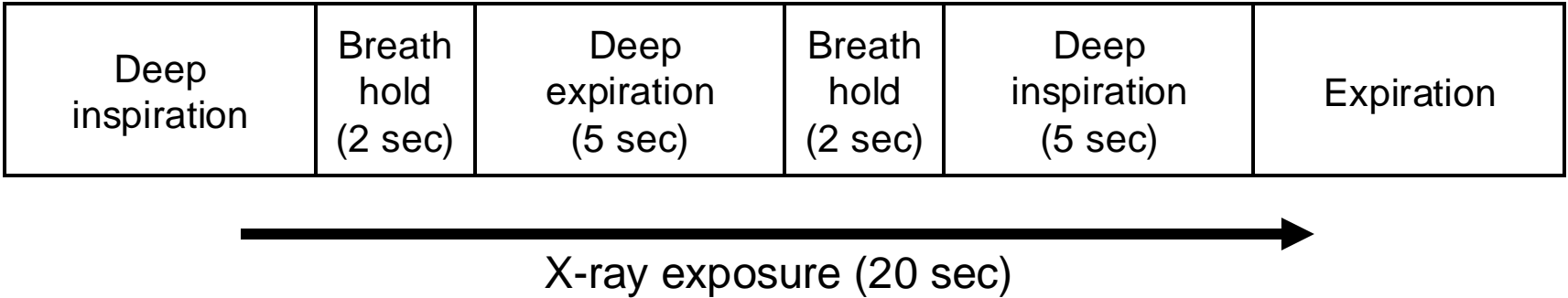

Supplement: Supplementary file 1 — Supporting Information [file ACM2-27-e70717-s001.zip › 2026-09352-sup-0003--S.pdf]

**sFigure 2**

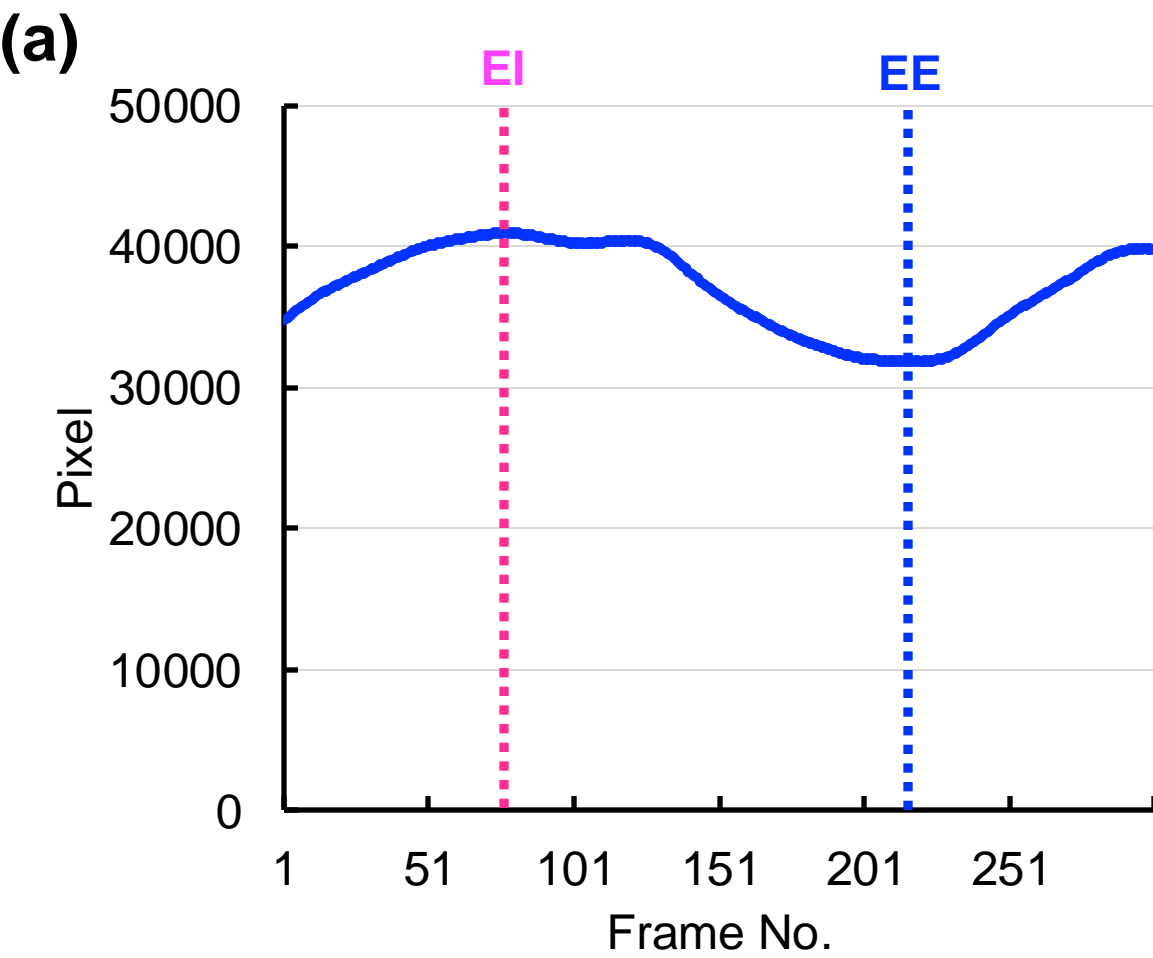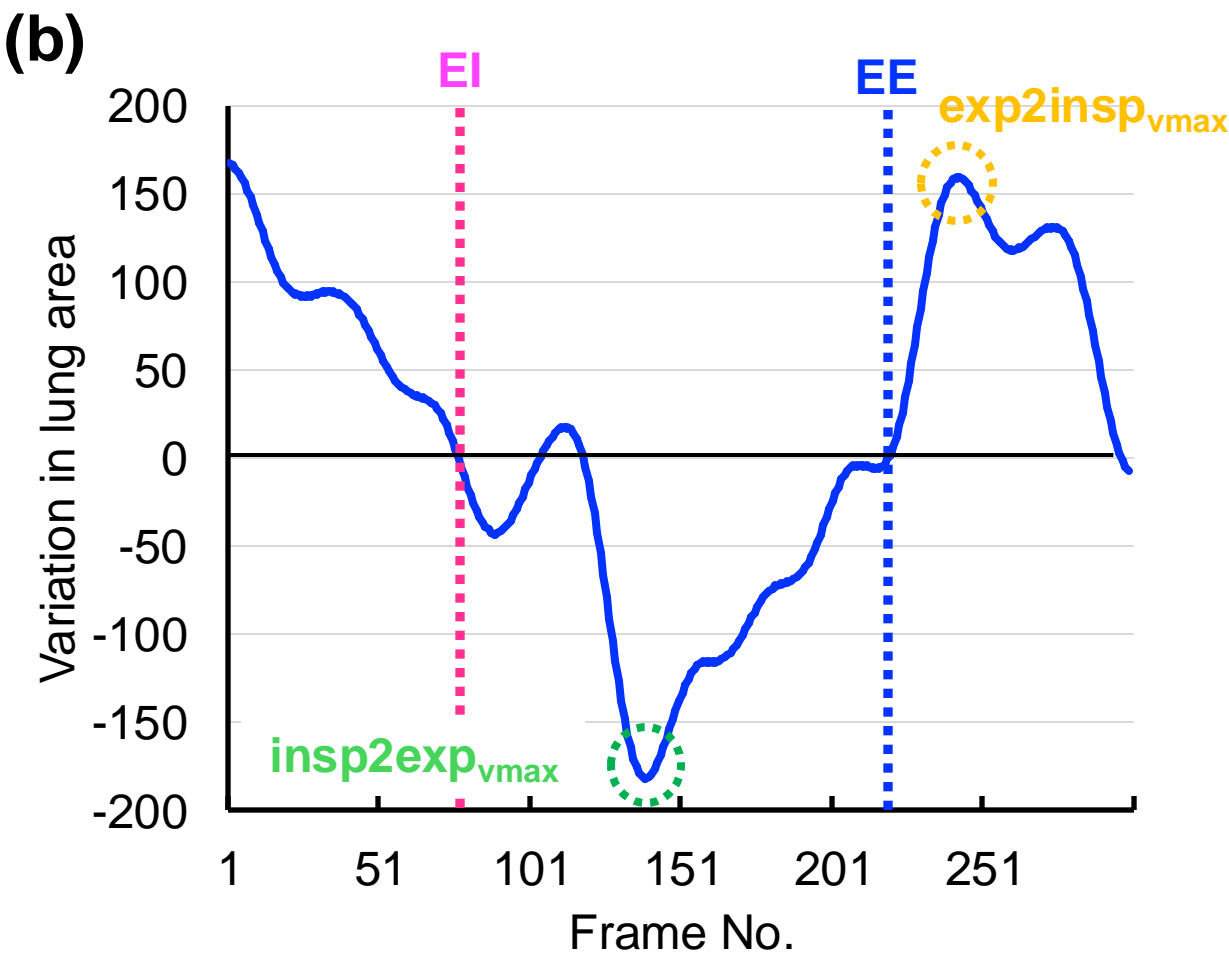

Supplement: Supplementary file 1 — Supporting Information [file ACM2-27-e70717-s001.zip › 2026-09352-sup-0004--S.pdf]
